# Inflammatory monocytes are detrimental to the host immune response during acute infection with *Cryptococcus neoformans*

**DOI:** 10.1371/journal.ppat.1007627

**Published:** 2019-03-21

**Authors:** Lena J. Heung, Tobias M. Hohl

**Affiliations:** 1 Infectious Diseases Service, Department of Medicine, Memorial Sloan Kettering Cancer Center, New York, New York, United States of America; 2 Immunology Program, Sloan Kettering Institute, Memorial Sloan Kettering Cancer Center, New York, New York, United States of America; University of Birmingham, UNITED KINGDOM

## Abstract

*Cryptococcus neoformans* is a leading cause of invasive fungal infections among immunocompromised patients. However, the cellular constituents of the innate immune response that promote clearance versus progression of infection upon respiratory acquisition of *C*. *neoformans* remain poorly defined. In this study, we found that during acute *C*. *neoformans* infection, CCR2^+^ Ly6C^hi^ inflammatory monocytes (IM) rapidly infiltrate the lungs and mediate fungal trafficking to lung-draining lymph nodes. Interestingly, this influx of IM is detrimental to the host, since ablating IM or impairing their recruitment to the lungs improves murine survival and reduces fungal proliferation and dissemination. Using a novel conditional gene deletion strategy, we determined that MHC class II expression by IM did not mediate their deleterious impact on the host. Furthermore, although ablation of IM reduced the number of lymphocytes, innate lymphoid cells, and eosinophils in the lungs, the effects of IM were not dependent on these cells. We ascertained that IM in the lungs upregulated transcripts associated with alternatively activated (M2) macrophages in response to *C*. *neoformans*, consistent with the model that IM assume a cellular phenotype that is permissive for fungal growth. We also determined that conditional knockout of the prototypical M2 marker arginase 1 in IM and deletion of the M2-associated transcription factor STAT6 were not sufficient to reverse the harmful effects of IM. Overall, our findings indicate that *C*. *neoformans* can subvert the fungicidal potential of IM to enable the progression of infection through a mechanism that is not dependent on lymphocyte priming, eosinophil recruitment, or downstream M2 macrophage polarization pathways. These results give us new insight into the plasticity of IM function during fungal infections and the level of control that *C*. *neoformans* can exert on host immune responses.

## Author summary

*Cryptococcus neoformans* is a fungus that is prevalent throughout the environment and can cause a fatal infection of the central nervous system when inhaled into the lungs by patients with impaired immune systems. Our understanding of the immune responses that either help clear *C*. *neoformans* from the lungs or permit development of disease remains limited. In this study, we used a mouse model of lethal *C*. *neoformans* infection to determine that inflammatory monocytes, immune cells that are often among the first responders to infections, actually facilitate the progression of infection rather than clearance. These findings establish a foundation for future work to target the immune response of inflammatory monocytes as a strategy to improve the outcomes of patients that develop *C*. *neoformans* infections.

## Introduction

The ubiquitous encapsulated yeast *Cryptococcus neoformans* causes invasive fungal infections in immunocompromised patients, particularly those with AIDS, solid organ transplants, and cancer [1]. Even with optimal combination antifungal therapy, cryptococcosis has a high morbidity and mortality rate [2, 3]. Since *C*. *neoformans* enters the respiratory tract before disseminating to the central nervous system [1], defining the cellular and molecular mechanisms of the pulmonary innate immune response is critical for the development of novel treatment options that can promote fungal sterilization in the lungs.

C-C chemokine receptor 2 (CCR2)- and Ly6C^hi^-expressing inflammatory monocytes (IM) and their derivatives, including macrophages and dendritic cells (DCs), exhibit beneficial roles in innate host defense against many fungal pathogens, including *Aspergillus fumigatus* [4, 5], *Blastomyces dermatitidis* [6], *Candida albicans* [7], and *Histoplasma capsulatum* [6, 8]. For example, during pulmonary aspergillosis, IM directly engage and kill fungal cells, regulate innate immune activation of neutrophils, and facilitate adaptive CD4^+^ T cell responses [4, 5].

The role of IM in innate immunity to acute infection with *C*. *neoformans* has not been systematically examined. In models of subacute and chronic pulmonary cryptococcosis, there is an initial fungal expansion phase that is followed by prolonged, but largely progressive, fungal clearance through CD4^+^ and CD8^+^ T cell-dependent mechanisms [9, 10]. During the expansion phase, CCR2-dependent signals mediate the accumulation of DCs and macrophages in the lungs [11–13]. The latter express inducible nitric oxide synthase (NOS2) and tumor necrosis factor (TNF) and appear to act in a fungicidal manner in vitro [13]. Defective CCR2 signaling impairs fungal clearance and correlates with the development of T helper 2 (Th2) cytokine-dominated responses [14, 15]. More recent work indicates that IM and macrophages may play important roles in protective immune responses generated by candidate vaccine strains of *C*. *neoformans* [16–19]. These data suggest that IM and their derivatives may play beneficial roles in innate immunity during subacute and chronic cryptococcal infections.

On the other hand, in a lethal model of acute cryptococcosis, it has been observed that enhanced IM accumulation in the lungs correlates with decreased survival [20]. It is not known if IM may promote progressive infection by specific effects on fungal growth or T helper responses or if IM influx is part of a general inflammatory response in the lungs during acute cryptococcal infection. *C*. *neoformans* is also a facultative intracellular pathogen that has been shown in vitro to replicate within monocytes and macrophages and exit via non-lytic exocytosis [21–24]. Thus, it has been proposed that these cells can function as “Trojan horses” that facilitate fungal proliferation and dissemination [25–27]. Together, these data support an alternative model in which IM could be detrimental in the host response to acute *C*. *neoformans* infection.

In this study, we sought to clarify the role of IM and their derivatives in a murine model of acute cryptococcosis using a highly virulent serotype A strain of *C*. *neoformans*. We utilized CCR2-DTR depleter mice [5] and a new constitutive CCR2-Cre mouse model to probe the functional role of IM in *C*. *neoformans* control and host survival. Interestingly, we found that in the absence of IM, murine survival is improved and there is decreased fungal burden in the lungs and disseminated sites, indicating that IM are harmful for host anti-cryptococcal immunity. We did not find experimental evidence that immunopathology or cellular crosstalk between IM and lymphocytes or eosinophils influence these infectious outcomes. However, we observed that IM in the lungs exhibit an alternatively activated (M2)-like macrophage transcriptional profile in response to *C*. *neoformans*. In particular, IM significantly upregulated expression of the gene encoding arginase 1 (ARG1), an M2 marker that may modulate immune responses due to its competition for L-arginine substrate with NOS2, a marker for classically activated (M1) macrophages [28–30]. M2 macrophages have previously been demonstrated to have decreased anti-cryptococcal activity against *C*. *neoformans* in vitro compared to classically activated (M1) macrophages [31]. Interestingly, the conditional knockout of *Arg1* in IM and the deletion of STAT6, a transcriptional regulator of *Arg1* [32, 33], in hematopoietic cells could not reverse the impact of IM on host outcomes during acute cryptococcosis. In summary, our study defines a novel cell-intrinsic role for IM as mediators of detrimental host immune responses to a respiratory fungal pathogen and indicates that the subversion of these potential antifungal effector cells by *C*. *neoformans* occurs early in the IM response.

## Results

### IM recruited to the lungs worsen infectious outcomes in an acute respiratory infection model of murine cryptococcosis

**Fig 1:**
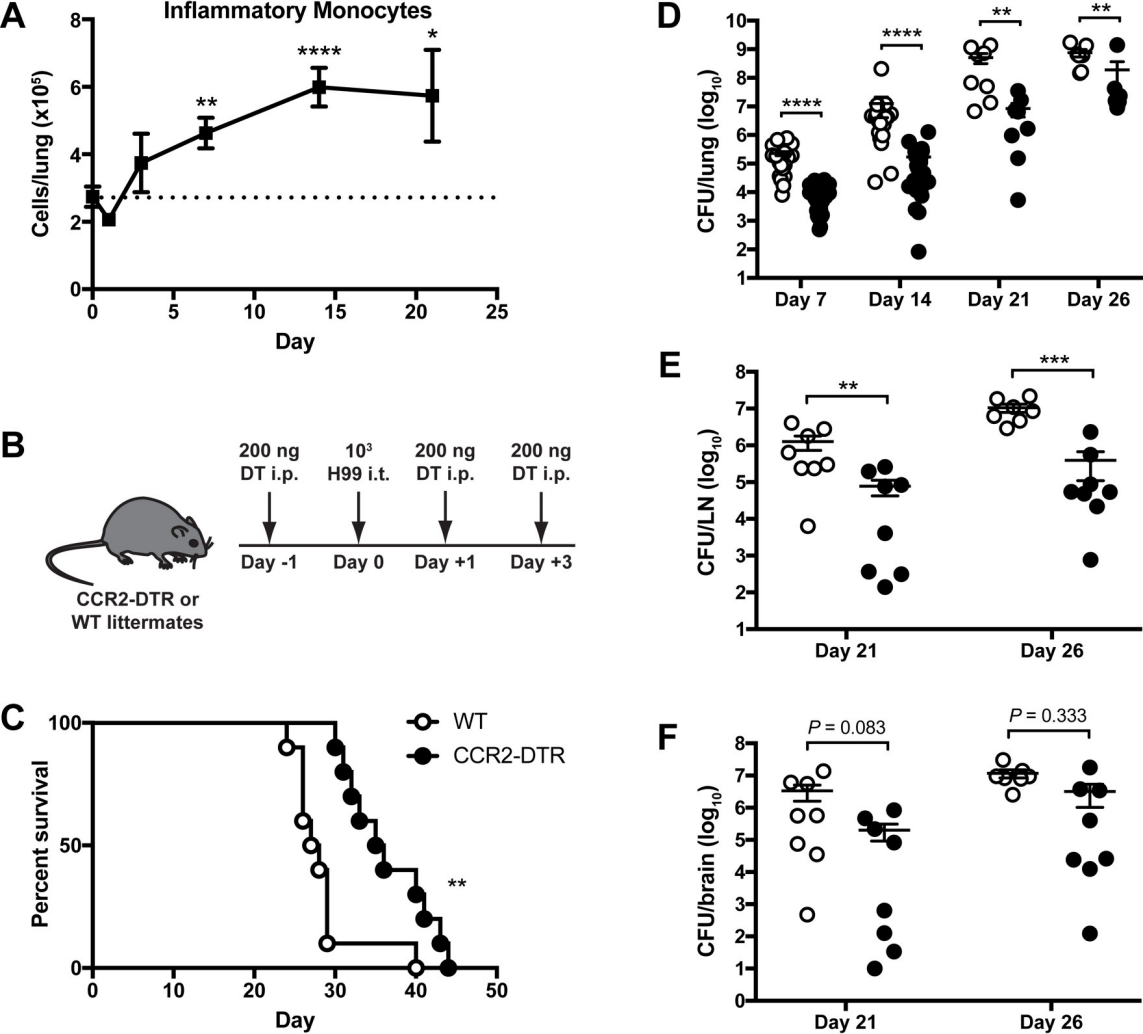
Inflammatory monocytes promote detrimental host responses to *Cryptococcus neoformans*. (A) IM in the lungs of C57BL/6 (WT) mice after intratracheal (i.t.) challenge with *C*. *neoformans* strain H99 relative to naive mice (dotted line). Data were pooled from eight independent experiments (*n* = 6–26 total mice per timepoint). (B) Diphtheria toxin (DT) was administered intraperitoneally (i.p.) as illustrated to ablate IM in CCR2-DTR mice. (C) Kaplan-Meier survival curve of WT littermate controls (white circles) and CCR2-DTR mice (black circles) after administration of DT and H99. Data were pooled from two independent experiments (*n* = 10 total mice per group). (D-F) CFU in (D) lung, (E) mediastinal lymph node (LN), and (F) brain homogenates from WT and CCR2-DTR mice at indicated timepoints. Data were pooled from nine independent experiments (*n* = 7–27 total mice per group per timepoint). *, *P* < 0.05. **, *P* < 0.01. ***, *P* < 0.001. ****, *P* < 0.0001.

Our studies utilized an acute infection model in which C57BL/6 mice were administered 10^3^ yeast cells of *C*. *neoformans* serotype A strain H99 intratracheally (i.t.). This infection model was uniformly fatal with a range of inocula from 10^2^ to 10^5^ yeast cells (S1A Fig). We found that IM accumulated in the lungs of mice within the first week after respiratory challenge and persisted during the course of progressive infection (Fig 1A). There was also an increase in pulmonary CD11b^+^ DCs and macrophages during infection (S1B Fig), consistent with the known developmental relationships between lung-infiltrating IM and these immune cell subsets during fungal infections [5, 12, 13].

To determine the role of IM at the onset of infection, these cells were transiently ablated in CCR2-DTR mice [5] by administering intraperitoneal (i.p.) diphtheria toxin (DT) on days -1, +1 and +3 relative to infection with H99 (Fig 1B). DT treatment of CCR2-DTR mice also resulted in significant decreases in DCs and macrophages in the lungs on day 7 post-infection (p.i.) (S2 Fig). DCs in CCR2-DTR mice returned in numbers comparable to non-transgenic littermate controls (WT) by day 14 p.i., while macrophages exhibited a slower return toward WT levels (S2 Fig). Compared to WT mice, CCR2-DTR mice had significantly prolonged survival (median survival 35.5 days for CCR2-DTR versus 27.5 days for WT) (Fig 1C) and decreased fungal burden in the lungs on days 7, 14, 21 and 26 p.i. (Fig 1D). CCR2-DTR mice had a similar ~1 log reduction in lung fungal burden compared to WT littermates when challenged with a 10-fold higher inoculum of 10^4^ H99 yeast cells (S3A Fig) or with 10^4^ yeast cells of a less virulent *C*. *neoformans* serotype D strain 52D (S3B Fig). The lungs of WT mice were grossly enlarged compared to those of CCR2-DTR mice (S4A Fig), which may be due to the observed differences in fungal burden as well as a trend toward increased pulmonary infiltrates in WT mice (S4B–S4D Fig). Histopathology analysis found that multinucleated giant cells (MGC) contain phagocytized fungal organisms multifocally in the lungs of both WT and CCR2-DTR mice (S4C–S4D Fig). However, there were more extracellular fungal organisms and fewer macrophages and MGC in the CCR2-DTR lungs compared to the WT lungs (S4C–S4D Fig). CCR2-DTR mice also had a decreased fungal burden in the mediastinal lymph node (Fig 1E) and a trend toward a decreased fungal burden in the brain (Fig 1F) on days 21 and 26 p.i. compared to WT mice. These results suggest that IM and their derivatives are harmful to the host during acute cryptococcosis by promoting fungal proliferation and dissemination from the lungs.

**Fig 2:**
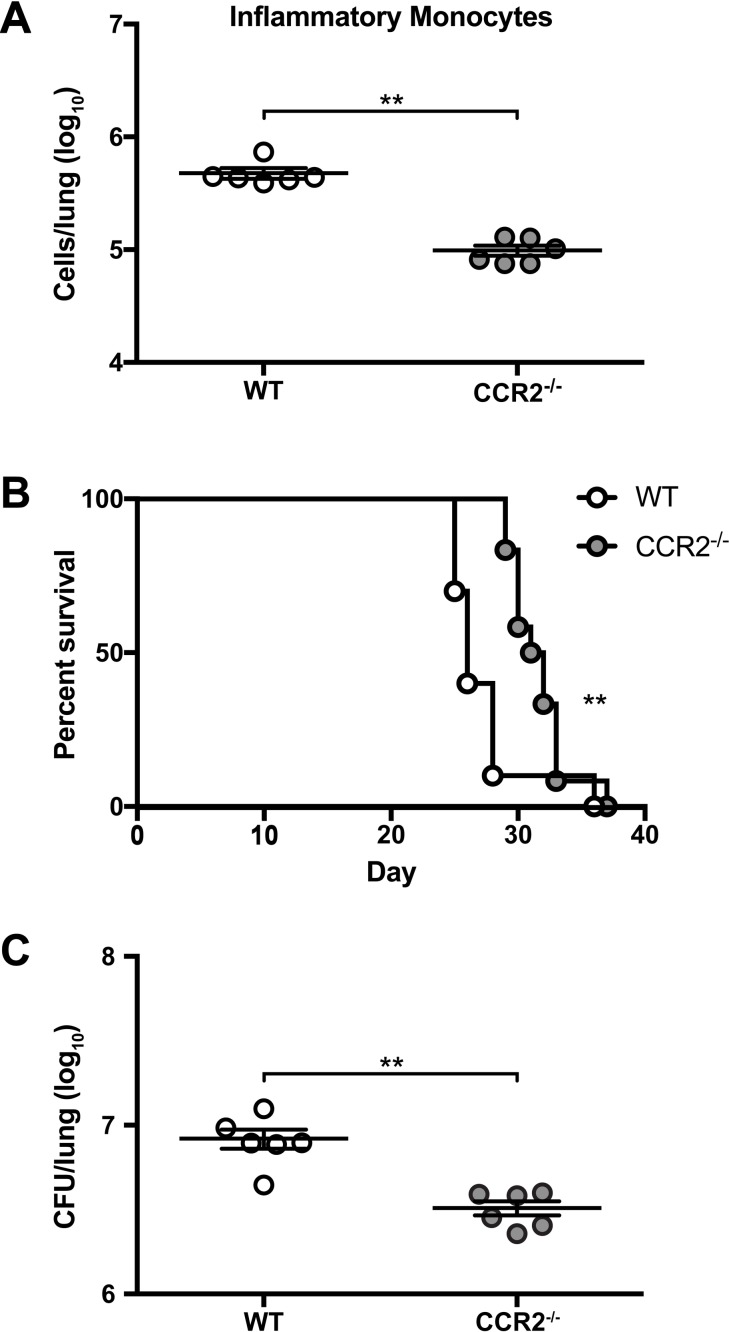
Trafficking of inflammatory monocytes to the lungs regulates detrimental immune responses. (A) IM in the lungs of WT mice (white circles) and CCR2^-/-^ mice (gray circles) on day 14 after i.t. challenge with H99. Data are from one experiment (n = 6 mice per group). (B) Kaplan-Meier survival curve of WT and CCR2^-/-^ mice challenged with H99. Data were pooled from two independent experiments (*n* = 10–12 total mice per group). (C) CFU in the lungs of WT and CCR2^-/-^ mice on day 14 p.i. Data are from one experiment (*n* = 6 mice per group). **, *P* < 0.01.

To examine whether the recruitment of IM to the lungs promotes detrimental responses to *C*. *neoformans*, we utilized CCR2^-/-^ mice [34], in which the ability of monocytes to migrate out of the bone marrow (BM) is significantly impaired, resulting in an ~75% reduction in circulating monocytes under homeostatic conditions [35]. We confirmed that CCR2^-/-^ mice have an ~79% decrease in the number of IM in the lungs compared to WT mice 14 days after *C*. *neoformans* challenge (Fig 2A). CCR2^-/-^ mice demonstrated a significant survival benefit (median survival 31.5 days for CCR2^-/-^ versus 26 days for WT) (Fig 2B) and a lower lung fungal burden on day 14 p.i. (Fig 2C) compared to WT mice. Collectively, these data indicate that the initial recruitment of IM to the lungs plays a key role in mediating harmful outcomes during acute cryptococcosis. Consistent with this model, IM depletion later in the course of infection, by administering DT on days +6, +8, and +10 p.i. to CCR2-DTR mice, did not give rise to any differences in survival between CCR2-DTR and WT mice (S3C Fig).

### CCR2-Cre mice enable conditional knockout of genes in IM

**Fig 3:**
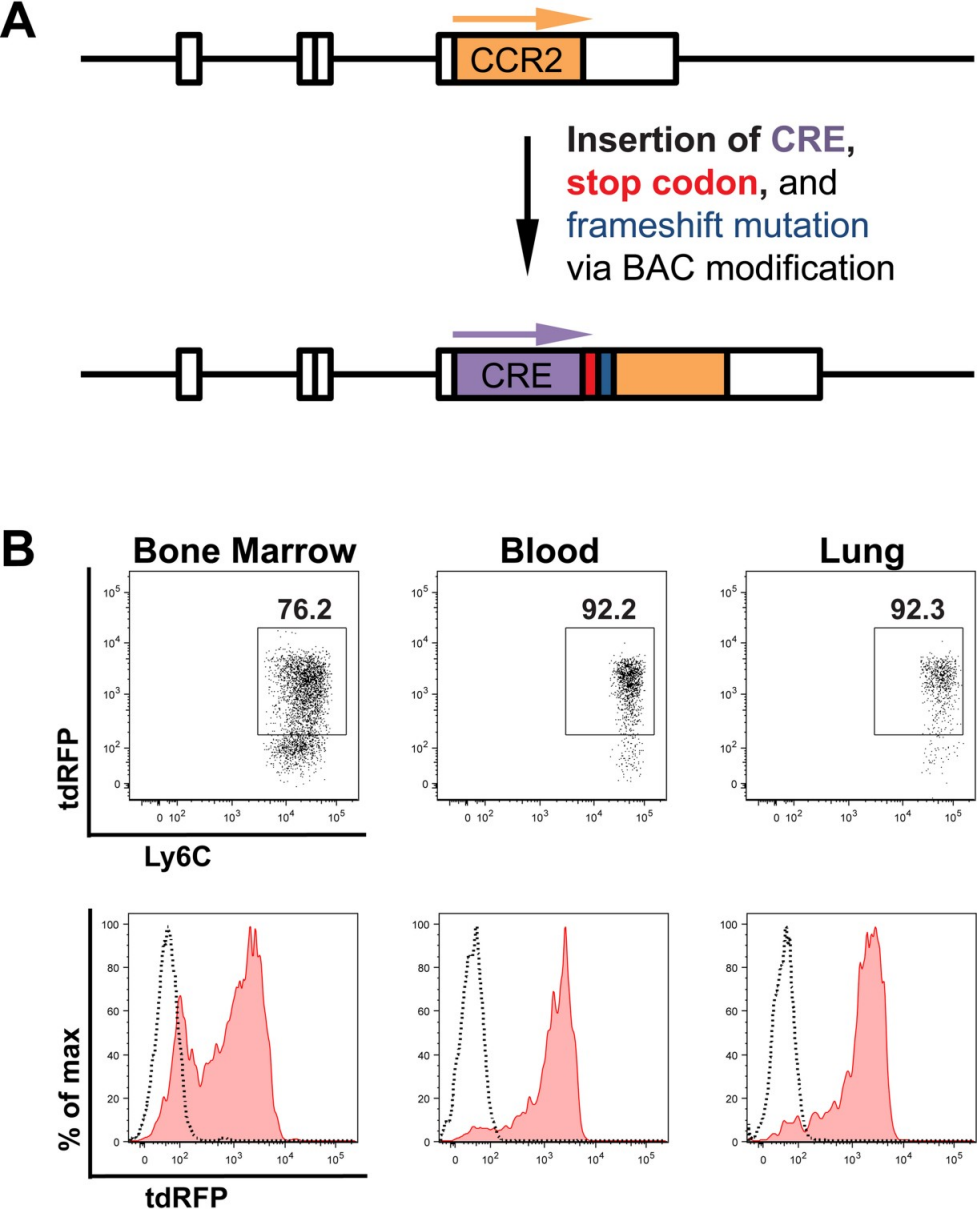
Generation and validation of a CCR2-Cre mouse. (A) Schematic of the modification of a BAC containing the endogenous CCR2 locus in order to insert the Cre recombinase gene, a stop codon, and a frameshift mutation downstream of the CCR2 promoter. (B) Representative flow plots and histograms of tdRFP expression by IM in the bone marrow, blood and lung of naive CCR2-Cre Rosa26^flSTOP-tdRFP^ mice. The dotted lines in the histograms represent naive Rosa26^flSTOP-tdRFP^ control mice.

To establish a strategy to investigate the role of IM functions in our model, we generated a CCR2-Cre mouse that allows us to conditionally knockout genes of interest in IM. We used a bacterial artificial chromosome (BAC) transgenic approach to introduce the Cre recombinase gene downstream of the CCR2 promoter (Fig 3A and S5A Fig). We identified four potential CCR2-Cre founder mice and evaluated the efficiency and specificity of Cre expression in these mice by crossing them to Rosa26^flSTOP-tdRFP^ mice [36]. The founder mouse selected to establish the CCR2-Cre colony had excellent expression of tdRFP in monocytes in all tissues analyzed, including ~90% of monocytes in the blood and lungs (Fig 3B and S5B–S5G Fig).

### IM do not regulate lymphocyte responses during acute cryptococcosis

**Fig 4:**
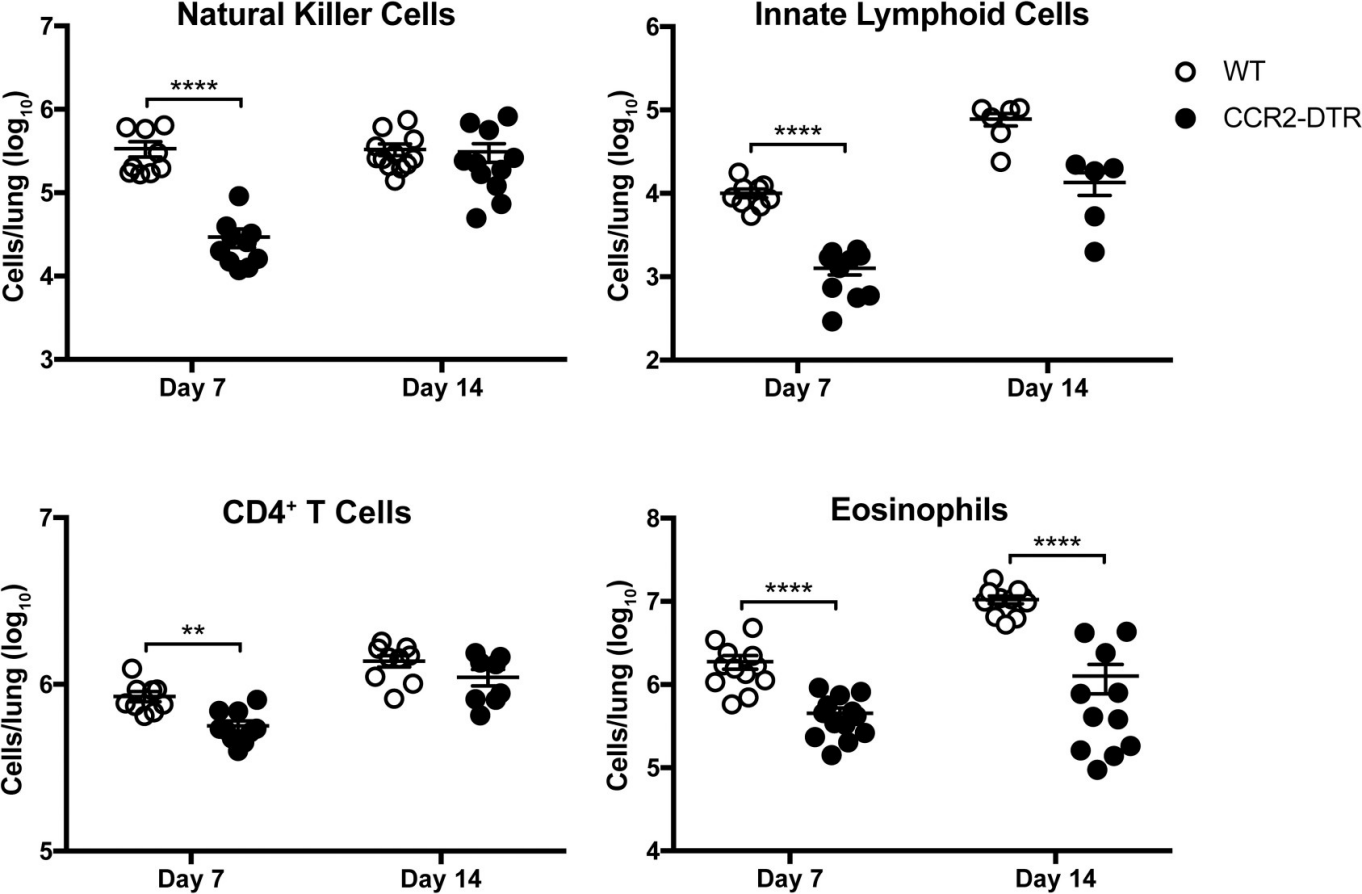
Inflammatory monocytes regulate the presence of other immune cells in the lungs. Natural killer cells, innate lymphoid cells, CD4^+^ T cells, and eosinophils in the lungs of WT mice (white circles) and CCR2-DTR mice (black circles) challenged with H99 were ennumerated on days 7 and 14 p.i. Data were pooled from four independent experiments (*n* = 5–14 total mice per group). **, *P* < 0.01. ****, *P* < 0.0001.

Ablation of IM led to a reduction in the number of pulmonary lymphocytes, including natural killer (NK) cells, innate lymphoid cell subsets (ILCs), and CD4^+^ T cells on day 7 p.i. (Fig 4), suggesting that IM may regulate lymphocyte activity against *C*. *neoformans*. We also found a marked reduction in the levels of the Th2 cytokines IL-5 and RANTES/CCL5 along with a slight decrease in TNF production at the same timepoint in IM-ablated CCR2-DTR mice (S6 Fig). These data indicated that IM could be deleterious because they facilitate the generation of harmful Th2 responses during acute cryptococcosis.

**Fig 5:**
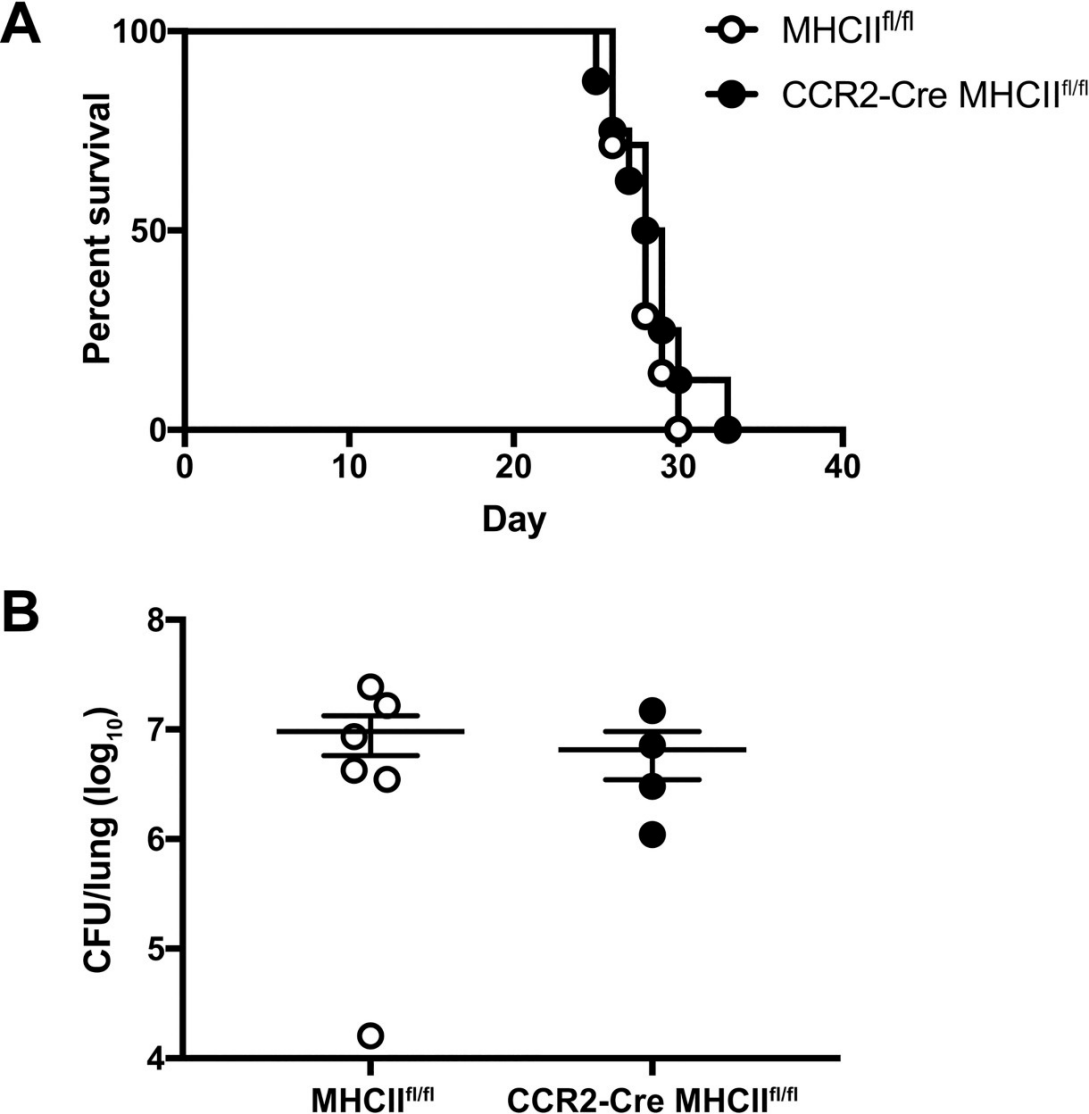
Inflammatory monocytes regulate detrimental immune responses to *C*. *neoformans* independent of MHCII expression. (A) Kaplan-Meier survival curve of MHCII^fl/fl^ control mice (white circles) and CCR2-Cre MHCII^fl/fl^ mice (black circles) challenged with H99. Data are from one experiment (*n* = 7–8 mice per group). (B) CFU in the lungs on day 14 p.i. Data are from one experiment (*n* = 4–6 mice per group).

To examine the possible link between MHC class II (MHCII) antigen presentation by IM and host Th2 responses, we generated CCR2-Cre MHCII^fl/fl^ mice to conditionally knockout MHCII expression in IM and their derivative cells. We confirmed the loss of MHCII expression in a subset of CD11b^+^CD11c^+^ cells, consistent with IM-derived DCs (S7 Fig). However, conditional knockout of MHCII in these cells had no effect on survival (Fig 5A) or lung fungal burden on day 14 p.i. (Fig 5B) compared to MHCII^fl/fl^ littermate controls. Therefore, MHCII antigen presentation by IM and their derivatives does not mediate harmful immune responses to *C*. *neoformans*.

**Fig 6:**
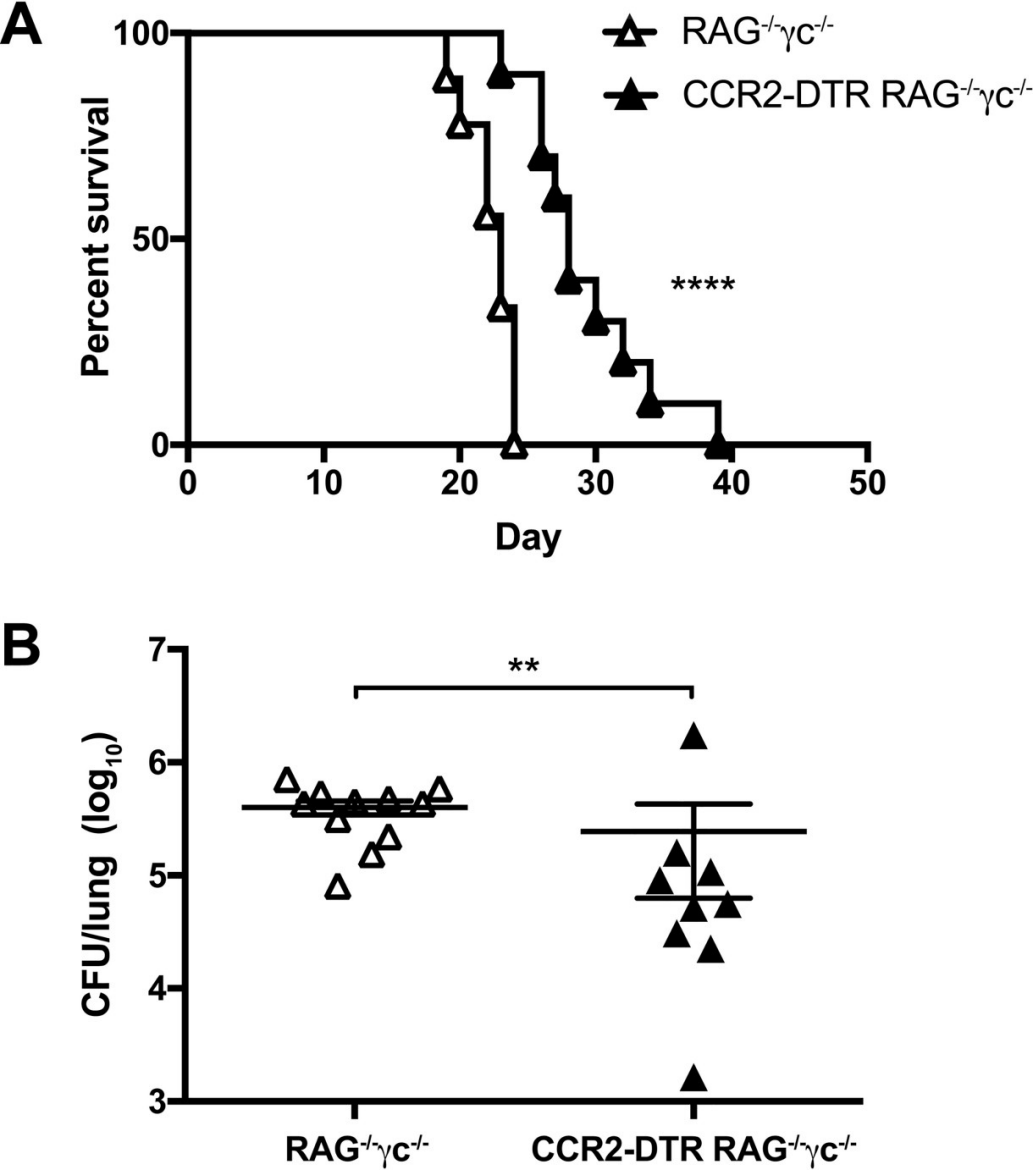
Lymphocytes are not essential for the detrimental effects of inflammatory monocytes. (A) Kaplan-Meier survival curve of RAG^-/-^γc^-/-^ control mice (white triangles) and CCR2-DTR RAG^-/-^γc^-/-^ mice (black triangles) challenged with H99. Data are from two independent experiments (*n* = 9–10 mice per group). (B) CFU in the lungs on day 7 p.i. Data are from two independent experiments (*n* = 9–11 mice per group). **, *P* < 0.01; ****, *P* < 0.0001.

To determine if lymphocytes are essential for IM-mediated outcomes, we next crossed the CCR2-DTR mice with RAG^-/-^γc^-/-^ mice, in which lymphocytes, including T- and B- cells, NK cells, and ILCs, are absent. These CCR2-DTR RAG^-/-^γc^-/-^ mice enabled DT-mediated ablation of IM in a lymphocyte-deficient background. We found that CCR2-DTR RAG^-/-^γc^-/-^ mice had improved survival compared to non-transgenic RAG^-/-^γc^-/-^ littermates (median survival of 28 days for CCR2-DTR RAG^-/-^γc^-/-^ versus 23 days for RAG^-/-^γc^-/-^) (Fig 6A) and a decreased lung fungal burden on day 7 p.i. (Fig 6B). Therefore, lymphocytes were not required to mediate the detrimental effects of IM in our model. We note that although IM are the most prevalent CCR2-expressing cells, subsets of T cells, NK cells, and ILC precursors can express variable levels of CCR2 [4, 37, 38]. Thus, these results confirm that IM, not other CCR2-expressing lymphocytes, were responsible for the phenotypes we observed.

### Eosinophils do not regulate host outcomes in acute cryptococcosis

**Fig 7:**
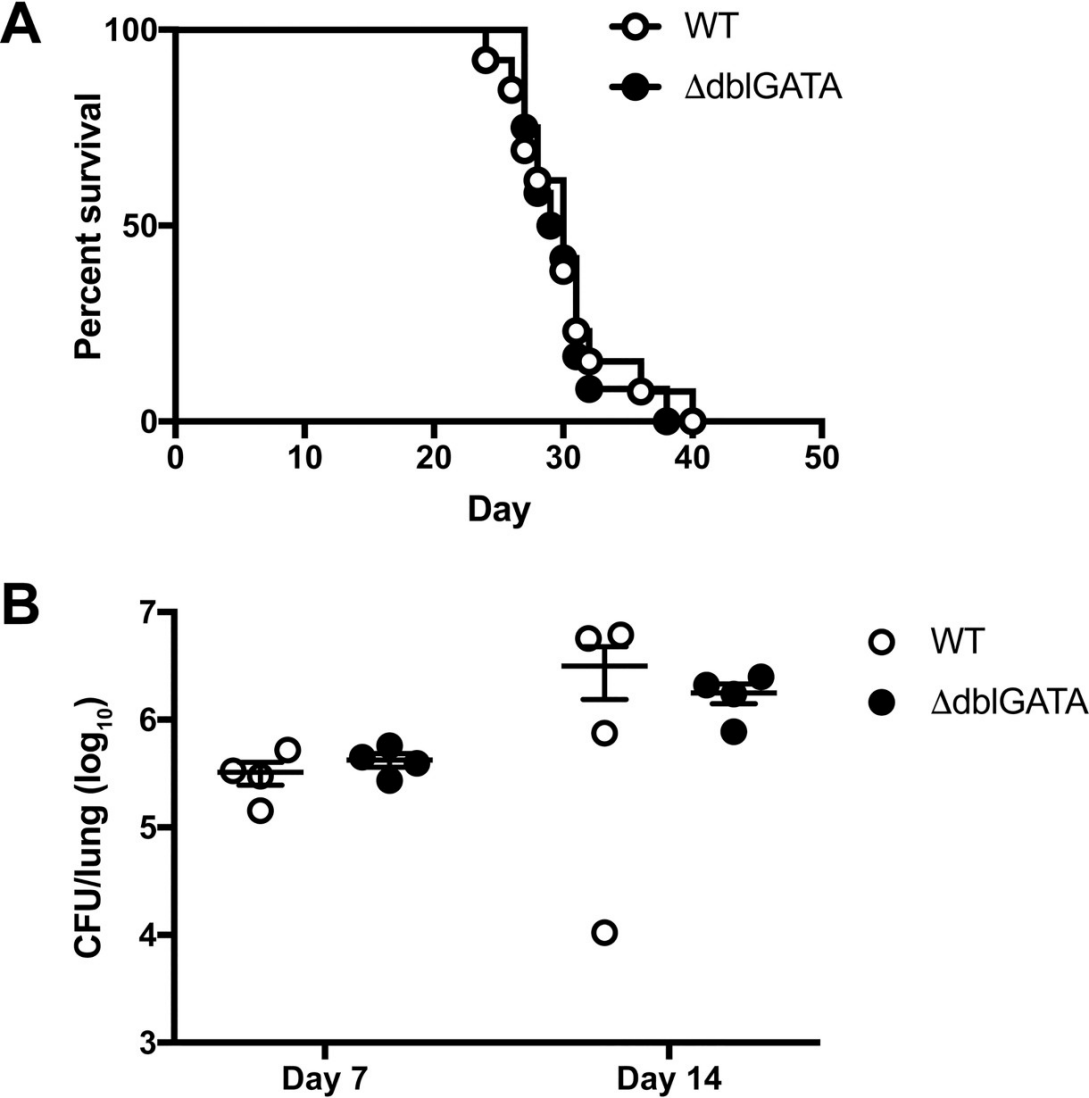
Eosinophils do not regulate infectious outcomes after *C*. *neoformans* challenge. (A) Kaplan-Meier survival curve of WT mice (white circles) or ΔdblGATA mice (black circles) challenged with H99. Data were pooled from two independent experiments (*n* = 12–13 total mice per group). (B) CFU in the lungs on days 7 and 14 p.i. Data were pooled from two independent experiments (*n* = 4 total mice per group).

Previous studies have suggested that eosinophils are associated with cryptococcal disease in humans and mice [39–48] and positively correlate with murine susceptibility to cryptococcosis [45, 49]. We observed a reduction in eosinophils on days 7 and 14 p.i. in the lungs of IM-ablated CCR2-DTR mice compared to WT littermates (Fig 4). Since the eosinophil-active cytokines IL-5 and RANTES/CCL5 were also diminished in CCR2-DTR mice (S6 Fig), we investigated whether eosinophils may mediate the downstream effects of IM. After respiratory challenge with *C*. *neoformans*, eosinophil-deficient ΔdblGATA mice [50] did not exhibit any differences in survival (Fig 7A) or lung fungal burden on days 7 and 14 p.i. (Fig 7B) compared to WT control mice. These findings indicate that pulmonary eosinophilia likely represents a byproduct of an ineffective, Th2-skewed immune response rather than a functional immune response to *C*. *neoformans*. Therefore, eosinophils are unlikely to mediate the harmful effects of IM.

### IM express M2 macrophage markers in response to respiratory *C*. *neoformans* challenge

**Fig 8:**
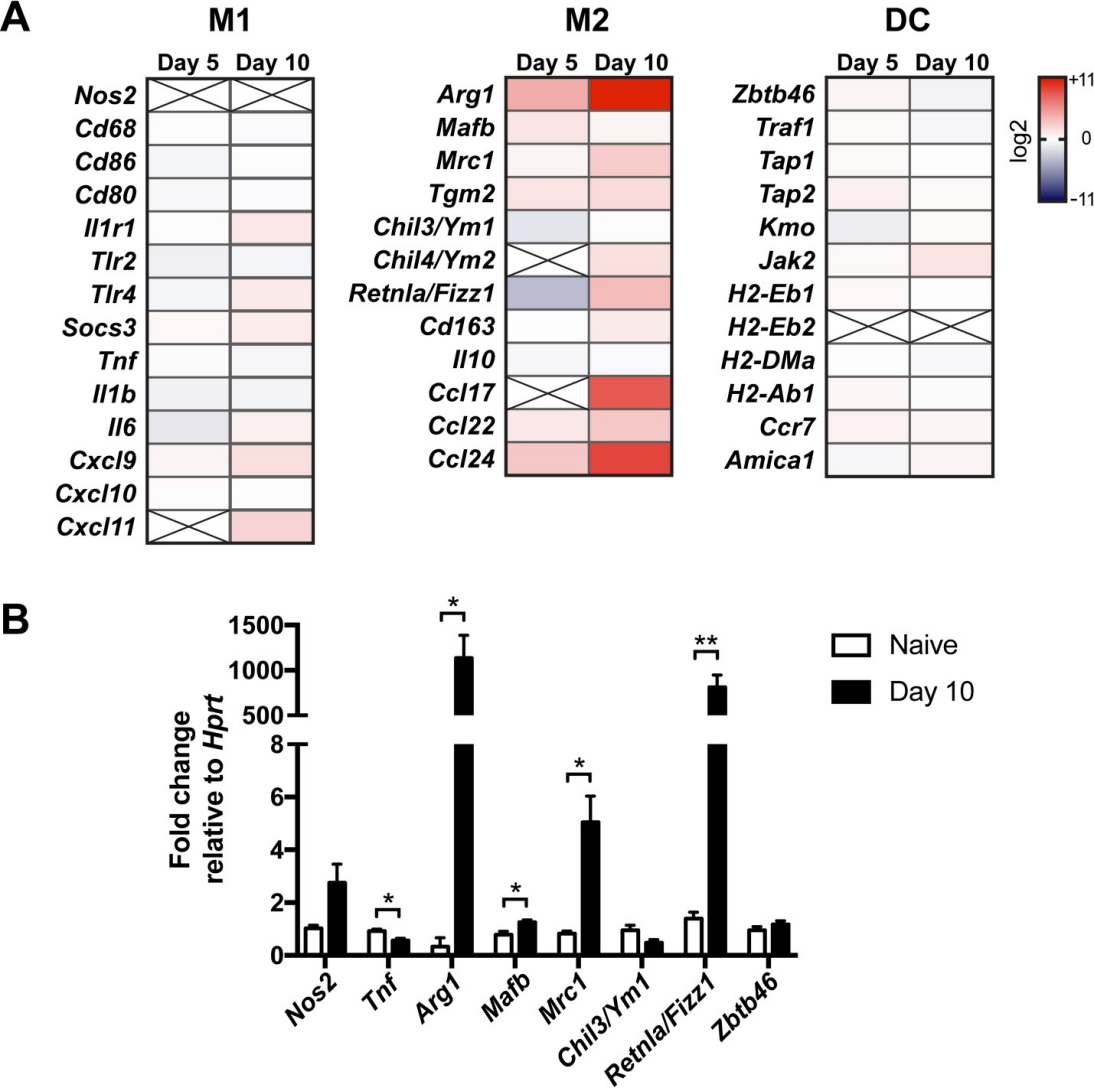
Inflammatory monocytes upregulate M2 macrophage markers in response to *C*. *neoformans*. (A) Heat maps of the expression of M1 macrophage, M2 macrophage, and dendritic cell (DC) markers in pulmonary IM on days 5 and 10 p.i. relative to IM from naive mice. X = not detected. Data are from one experiment (*n* = 6–7 mice per timepoint). (B) Quantitative RT-PCR of pulmonary IM from naive mice or mice on day 10 p.i. Data are from one experiment (*n* = 3 mice per group). **, *P* < 0.01 and *, *P* < 0.05 by *t*-test.

Since our data suggested that cellular crosstalk between IM and lymphocytes or eosinophils is not a primary mechanism by which IM regulate infectious outcomes, we next examined the role of direct intrinsic functions of IM in mediating detrimental immune responses to *C*. *neoformans*. RNASeq analysis of IM sorted from the lungs of naive and infected CCR2-GFP reporter mice [5] on days 5 and 10 p.i. was performed (Fig 8A). We found that, compared to naive IM, infected IM demonstrated significant increases (*P* adj < 0.05) in the transcription of genes commonly associated with an alternatively activated (M2) macrophage phenotype [51], including *Arg1*, mannose receptor C-type 1 (*Mrc1*/*Cd206*), transglutaminase 2 (*Tgm2*), resistin like alpha/found in inflammatory zone 1 (*Retnla*/*Fizz1*), and the chemokines *Ccl17* and *Ccl24*. Compared to naive IM, infected IM on day 10 p.i. also exhibited a high suppressor of cytokine signaling (*Socs*)*1*:*Soc3* ratio, that has been associated with the M2 phenotype [52, 53]. There was no differential expression of the M2 marker chitinase-like 3 (*Chil3*/*Ym1*). Among transcripts associated with classically activated (M1) macrophages, *Nos2* was not detected by RNASeq, and, except for a slight increase in the chemokine *Cxcl9* on day 10 p.i. (*P* adj < 0.05), there was no differential expression of the remaining transcripts. Among DC-associated transcripts, there was a slight increase in *Jak2* on day 10 p.i. (*P* adj < 0.05) but no changes in other transcripts, including those of the zinc finger and BTB domain containing 46 transcription factor (*Zbtb46*) and MHC class II molecules (*H2-Eb1*, *H2-Eb2*, *H2-DMa*, *H2-Ab1*).

The RNASeq findings were validated by qRT-PCR in naive and infected mice on day 10 p.i. (Fig 8B). We confirmed there was no significant change in the M1 marker *Nos2*, but we did detect a slight decrease in expression of *Tnf* in infected IM. We again saw significant increases in the M2 markers *Arg1*, *Mrc1* and *Retnla*/*Fizz1* and no change in *Chil3*/*Ym1* transcription, but we also detected an increase in the V-maf musculoaponeurotic fibrosarcoma oncogene homolog B transcription factor (*Mafb*), that promotes macrophage differentiation [54]. Finally, we confirmed no change in the DC marker *Zbtb46*. Together, these results suggest that *C*. *neoformans* subverts IM to assume an M2 macrophage-like phenotype that may be more permissive for fungal proliferation.

### Disruption of M2 polarization pathways does not reverse the harmful effects of IM

**Fig 9:**
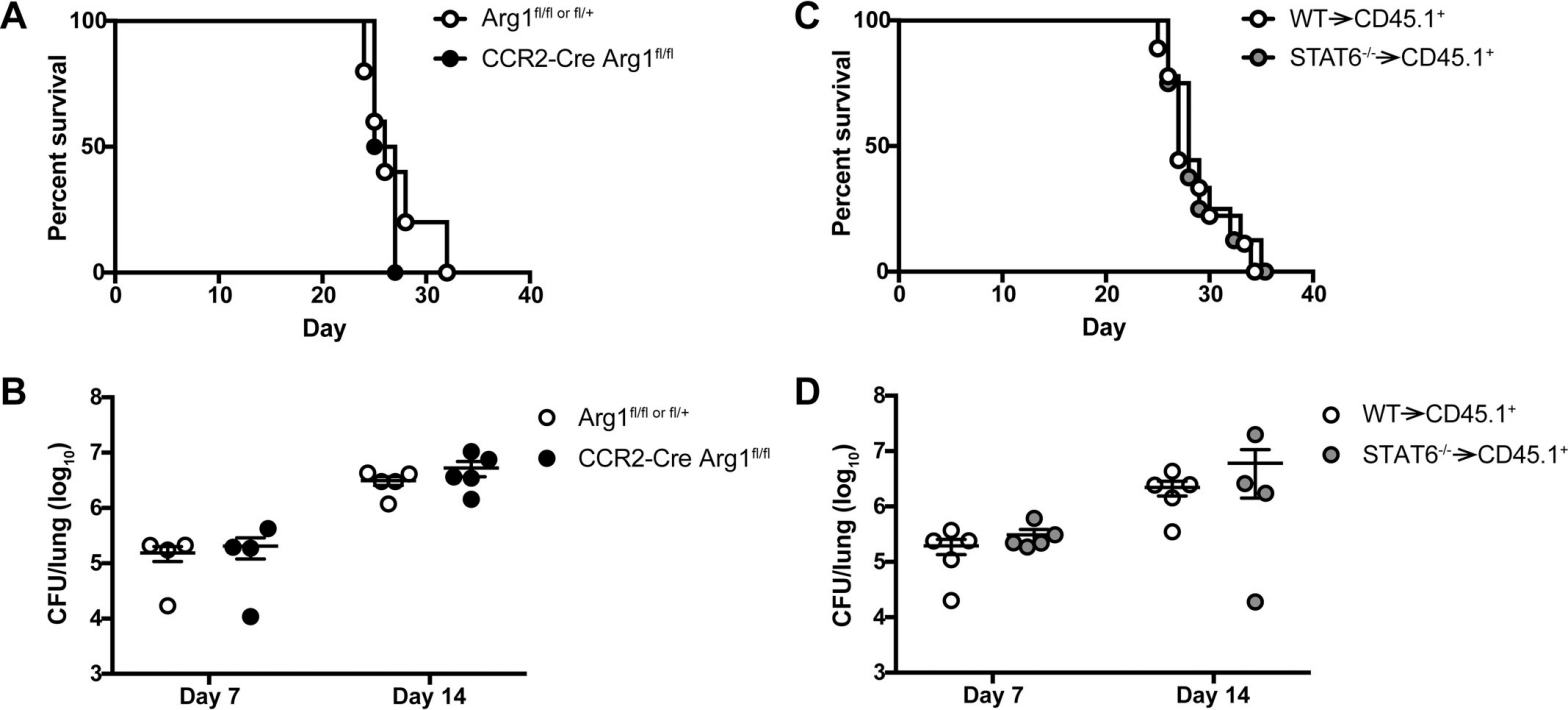
Blocking M2 macrophage polarization pathways does not reverse the detrimental effects of inflammatory monocytes. (A) Kaplan-Meier survival curve of control Arg1^flfl or fl/+^ mice (white circles) and CCR2-Cre Arg1^flfl^ mice (black circles). Data are from one experiment (*n* = 4–5 mice per group). (B) CFU in the lungs of Arg1^flfl or fl/+^ and CCR2-Cre Arg1^flfl^ mice on days 7 and 14 p.i. Data are from two experiments (*n* = 4–5 mice per group). (C) Kaplan-Meier survival curve of control WT→ CD45.1^+^ bone marrow chimeras (white circles) and STAT6^-/-^ → CD45.1^+^ bone marrow chimeras (gray circles). Data are pooled from two experiments (*n* = 8–9 total mice per group). (D) CFU in the lungs of WT→ CD45.1^+^ and STAT6^-/-^→ CD45.1^+^ bone marrow chimeras on days 7 and 14 p.i. Data are from two experiments (*n* = 4–5 mice per group).

*Arg1*, an archetypal M2 macrophage marker, was one of the most highly expressed transcripts in infected IM (Fig 8). Previous studies indicate that ARG1 may compete with the M1 macrophage marker NOS2 for L-arginine substrate that it metabolizes into urea and L-ornithine, thereby reducing nitric oxide production by NOS2 [28–30]. Since M2 macrophages are less fungicidal than M1 macrophages against *C*. *neoformans* in vitro [31], we investigated the potential role of IM-intrinsic ARG1 activity in the response to *C*. *neoformans* in the lungs by generating CCR2-Cre Arg1^fl/fl^ mice. Our results showed that conditional knockout of *Arg1* in IM decreased total arginase activity in the lungs by approximately 50% (S8A Fig) but did not have any effect on survival (Fig 9A) or lung fungal burden on days 7 and 14 p.i. (Fig 9B). Additionally, we observed that Vav1-Cre Arg1^fl/fl^ mice, that lack *Arg1* in all hematopoietic cells, had no difference in survival compared to Arg1^fl/fl or fl/+^ littermate controls (S8B Fig). Therefore, although infected IM highly express *Arg1*, it does not appear that *Arg1* mediates the detrimental effects of IM or other hematopoietic cells in response to *C*. *neoformans* challenge.

STAT6 is a transcription factor that mediates M2 macrophage polarization in the context of IL-4 and IL-13 signaling by regulating expression of M2 macrophage markers, including *Arg1*, *Mrc1*, and *Retnla*/*Fizz1* [55, 56]. To determine if more global blockade of M2 macrophage polarization pathways would reverse the detrimental host outcomes in our model of cryptococcosis, we generated STAT6^-/-^ BM chimeras to knockout STAT6 in hematopoietic cells, since STAT6^fl/fl^ mice are not commercially available. There was no difference in overall survival (Fig 9C) and lung fungal burden on days 7 and 14 p.i. (Fig 9D) in STAT6^-/-^ BM chimeras compared to control mice. Therefore, blocking STAT6-mediated pathways in hematopoietic cells, including IM, does not appear to be sufficient to counteract the harmful effects of IM during acute cryptococcosis. These results suggest that STAT6-regulated M2 markers, while associated with this macrophage phenotype, do not play an active role in macrophage function during acute cryptococcosis and that the subversion of IM function by *C*. *neoformans* more likely occurs at an earlier stage in the host-pathogen interaction.

## Discussion

In this study, we establish a critical role for IM in mediating detrimental host outcomes in a model of fatal respiratory infection with *C*. *neoformans*. These findings contrast with the beneficial functions of IM described in murine models of subacute and chronic pulmonary cryptococcosis and other fungal infections [4–8, 11, 14, 15] but align with studies that suggest IM and their derivatives are associated with progression of infection by *C*. *neoformans* [20–27]. The studies on IM during subacute and chronic cryptococcosis utilized CCR2^-/-^ mice on a BALB/c or mixed C57BL/6 and 129 background with the less virulent *C*. *neoformans* serotype D strain 52D [11, 14, 15]. We observed an improvement in lung fungal burden with ablation of IM in CCR2-DTR mice on the C57BL/6 background infected with 52D. However, Murdock et al. have shown that infection of C57BL/6 mice with the same inoculum of 52D i.t. is nonfatal up to 8 weeks p.i., suggesting this combination represents a more chronic model of infection [57]. Therefore, mouse genetic background may be an important factor in determining the role of IM in the immune response to *C*. *neoformans*, aside from the acuity of the infection, though we cannot yet exclude any contribution from differences in the fungal strains themselves based on available data. In any case, these disparate results indicate that IM possess a plasticity of function that can regulate the outcomes of infection and, thus, would make them an important target for immunomodulatory therapies against *C*. *neoformans*.

Accordingly, we sought to identify the mechanisms by which IM regulate infectious outcomes during cryptococcosis. To aid our investigation, we generated a CCR2-Cre transgenic mouse that demonstrates excellent Cre activity in IM, especially in the lungs. Along with the inducible *Ccr2*-creER^T2^ mouse previously generated by Becker and colleagues [58], the constitutive CCR2-Cre mouse expands the tools available to dissect IM function in a variety of immunologic processes.

One of our first observations was that IM contribute to Th2 cytokine production and regulate the presence of NK cells, ILCs, and CD4^+^ T cells in the lungs after *C*. *neoformans* challenge. IM and their macrophage and DC derivatives often play important roles in regulating lymphocyte responses to pulmonary infections through antigen presentation and chemokine secretion [5, 59, 60]. Interestingly, we found that neither conditional deletion of MHCII in IM nor lymphocyte deficiency affected IM-mediated infectious outcomes, suggesting that IM-lymphocyte crosstalk is not relevant for host immune responses during acute *C*. *neoformans* infection. The cellular source of IL-5 and RANTES/CCL5 in our model remains unclear. We did detect some transcription of RANTES/CCL5, but not IL-5, by IM in our RNASeq data. RANTES/CCL5 has been reported to be expressed by many cell types (immgen.org [61]) while the sources of IL-5 are typically Th2 cells and type 2 ILCs [62–64]. Thus, although the observed changes in these cytokines upon IM ablation may be related to direct secretion by IM, an alternative possibility is that differences in fungal burden influenced cytokine secretion by other immune cells, or a combination thereof.

We also investigated whether IM regulate the immune response to *C*. *neoformans* through the recruitment of eosinophils to the lungs. Some studies have suggested that eosinophils may play beneficial roles in rat models of cryptococcosis [46, 65, 66] or other fungal infections like aspergillosis [67–69]. However, eosinophilia has been correlated with poor outcomes in mouse and human cryptococcosis [39–49], and in the presence of IM in our model, we observed an upregulation of pulmonary IL-5 and RANTES/CCL5, cytokines that play important roles in eosinophil maturation and trafficking [70]. Ultimately, we found that eosinophil-deficient ΔdblGATA mice have no change in infectious outcomes compared to WT mice. It has also been previously reported that IL-5^-/-^ and eosinophil-deficient PHIL mice have little to no change in infectious outcomes during cryptococcosis, although the data was not formally shown [49]. Thus, our results confirm that eosinophils are a hallmark of the progression of cryptococcal infection, but do not appear, in and of themselves, to play an active role in mediating the outcomes of infection.

Since cellular crosstalk between IM and lymphocytes or eosinophils did not seem to play an important role in our model, we next investigated the intrinsic functions of IM that may facilitate progression of *C*. *neoformans* infection. Based on our transcriptional profiling of infected IM, it appears that these cells preferentially express markers of M2 macrophages in response to pulmonary challenge with *C*. *neoformans*. M2 macrophages are generally anti-inflammatory cells and are involved in tissue homeostasis or repair, though they can be phenotypically heterogenous [71]. It has previously been reported that *C*. *neoformans* may use monocytes and macrophages as protected reservoirs or “Trojan horses” that aid in fungal dissemination [21–25, 27] and that M2 macrophages are less fungicidal against *C*. *neoformans* than M1 macrophages [31]. Additionally, our histopathology analysis demonstrated that the lack of IM decreases the number of macrophages and multinucleated giant cells in the lung parenchyma and increases the incidence of extracellular fungal organisms in the lungs. Therefore, these results support the idea that exposure to *C*. *neoformans* renders IM and their macrophage derivatives permissive for fungal proliferation, so that their physical absence from the lungs can actually ameliorate infectious outcomes. It remains unclear whether IM directly facilitate dissemination of *C*. *neoformans* in this acute infection model or whether the higher lymph node and brain fungal burdens in WT mice are simply a reflection of the higher lung fungal burden in these mice compared to IM-ablated CCR2-DTR mice. However, studies on other fungal and bacterial infections have previously demonstrated the ability of IM to participate in direct transport of microbes from the lung to disseminated sites [5, 72, 73].

Next, we examined if the harmful effects of IM during acute cryptococcosis could be reversed by targeting M2 macrophage polarization pathways. *Arg1* is a known M2 marker that was markedly upregulated in infected IM. Previous studies indicate that the fungal pathogen *C*. *albicans* can suppress NOS2 activity and induce ARG1 activity in human macrophages, resulting in decreased NO production and improved fungal survival [74–77]. Our studies show that conditional knockout of *Arg1* in IM does not improve survival or lung fungal burden after *C*. *neoformans* challenge. Although it has been suggested that ARG1 and NOS2 can compete for the same pool of L-arginine substrate [29, 30, 78, 79], it may be that disrupting ARG1 activity and increasing L-arginine availability is not sufficient. A second, positive signal may also be required to induce NOS2 transcription and activity, although it would be important to avoid triggering an uncontrolled inflammatory reaction that could lead to harmful immunopathology. Other studies have also indicated that despite the association of *Arg1* with M2 macrophages, ARG1 may not always be a functional component of the immune response [80].

We subsequently considered whether the functional outcomes of M2 polarization may be controlled further upstream. Previous studies indicate that global deletion of IL-13, IL-4, and IL-4Rα, as well as conditional knockout of IL-4Rα in myeloid cells, improves murine outcomes after respiratory challenge with *C*. *neoformans* [81–83]. Therefore, we investigated the role of STAT6, a transcription factor stimulated by IL-4 and IL-13 that regulates the expression of several M2 markers including *Arg1* [55, 56]. Knockout of STAT6 in hematopoietic cells using BM chimeras resulted in similar survival and lung fungal burden as control mice. These results contrast with the worse survival of global STAT6^-/-^ mice infected with *C*. *neoformans* strain KN99α, an H99-derived strain, observed by Wiesner et al [64]. Since we used BM chimeras, it is possible that a radioresistant cell population is contributing to the phenotype observed by Wiesner et al or that differences between the parental H99 strain and KN99α could be in play. Nevertheless, our results indicate that STAT6 signaling in IM does not play an important role in our infection model and suggest that *C*. *neoformans* may suppress pro-inflammatory signals further upstream that would otherwise direct the differentiation of IM into fungicidal M1 macrophages. For example, we previously observed that mice deficient in the DAP12 signaling adapter have improved infectious outcomes after *C*. *neoformans* challenge and that DAP12-deficient macrophages have improved uptake and killing of *C*. *neoformans* in vitro [84]. Thus, DAP12-mediated pathways may be important targets for promoting a more beneficial, classically activated immune response to *C*. *neoformans*.

Additional unanswered questions about the role of IM during cryptococcosis remain. We do not yet know if IM may influence the function of other myeloid cells like neutrophils, the role of which remains unclear during *C*. *neoformans* infection (reviewed in [85]), or of non-myeloid cells, e.g., lung epithelial cells, that can coordinate innate immune responses to other fungal infections [86]. Given the potential influence of mouse genetic background and fungal strain on IM function, it may be important to evaluate not only host immune differences, but also fungal-specific factors like capsule composition and Titan cell formation that may alter host-pathogen interactions [87, 88].

In summary, our study establishes a novel role for IM as crucial arbiters of infectious outcomes during acute cryptococcosis. Unlike in other pulmonary and disseminated fungal infections [4–8], IM do not aid in host defense but rather are subverted by *C*. *neoformans* to maintain a passive state that can be harnessed by the fungus for replication and dissemination. Our work was assisted by the generation of a CCR2-Cre mouse that will facilitate continued mechanistic evaluation of IM function in cryptococcosis. Using genetic reprogramming to target pathways that result in classical activation of IM and aid in fungal clearance would validate the concept that immunomodulation can be developed as a new therapeutic approach to manage cryptococcal infections.

## Materials and methods

### Chemicals and reagents

Chemicals were from Sigma-Aldrich, cell culture reagents were from Life Technologies/Gibco, and microbiological culture media were from BD Biosciences unless otherwise noted. Arginase activity was measured using an Arginase Activity Assay Kit (Sigma). Antibodies for flow cytometry were purchased from BD Biosciences, eBioscience or Tonbo unless otherwise indicated. Restriction enzymes were from New England Biolabs.

### Mice

C57BL/6J (stock #000664), MHCII^fl/fl^ (stock #013181) [89], and Arg1^fl/fl^ (stock #008817) [90] mice were purchased from the Jackson Laboratory (JAX). CCR2^-/-^ mice (JAX stock #004999) [34] were generously provided by Dr. E Pamer (MSKCC). Rosa26^flSTOP-tdRFP^ mice [36] were generously provided by Dr. J. Sun (MSKCC) [91]. The ΔdblGATA mice [50] on a C57BL/6 background were generously provided by Dr. H. Rosenberg (NIH). Vav1-Cre mice (JAX stock #008610) [92] were generously provided by Dr. F Geissmann (MSKCC). RAG2^-/-^γc^-/-^ mice (stock #4111) [93, 94] were purchased from Taconic. CD45.1^+^ mice (stock #564) were purchased from Charles River Laboratories. The CCR2-DTR depleter mice and CCR2-GFP reporter mice were generated as previously described [5, 95]. All mouse strains were bred and housed in the Memorial Sloan Kettering Cancer Center’s (MSKCC) Research Animal Resource Center under specific pathogen-free conditions. Mice on the RAG^-/-^γc^-/-^ background were maintained on amoxicillin- and Vitamin E-containing chow. To ablate monocytes, CCR2-DTR or CCR2-DTR RAG^-/-^γc^-/-^ mice and their control littermates were injected intraperitoneally with 200 ng (10 ng/g body weight) of diphtheria toxin (List Biological Laboratories) every other day for three doses, starting the day before infection (Fig 1B), unless otherwise noted. All experiments were conducted using male and female mice at age 6–8 weeks with sex- and age-matched mice in experimental and control groups. Experiments with CCR2-DTR, CCR2-DTR RAG^-/-^γc^-/-^, CCR2-Cre and Vav1-Cre mice used littermate control mice that were weaned from the same litters and co-housed.

### Ethics statement

All animal studies were performed with approval from the MSKCC Institutional Animal Care and Use Committee under protocol 13-07-008 and were compliant with all applicable provisions established by the Animal Welfare Act and the Public Health Services (PHS) Policy on the Humane Care and Use of Laboratory Animals.

### Generation of the CCR2-Cre mouse

A bacterial artificial chromosome (BAC) containing the CCR2-Cre transgene was generated using the recombineering strategy developed by Heintz and colleagues [96, 97]. Briefly, the BAC clone RP23-182D4 containing the endogenous CCR2 locus [5] was obtained from the BACPAC Resource Center at the Children’s Hospital Oakland Research Institute (CHORI). A 970 bp fragment upstream and a 711 bp fragment downstream of the CCR2 start codon were amplified (see all primers in Table 1). The 987 bp Cre gene was amplified from the CreER^t2^ frt Kan(R) frt plasmid [98], kindly provided by Dr. T. Buch (Univ. of Zurich). Overlap PCR was performed with these three fragments to generate a 2668 bp CCR2-Cre recombination cassette, in which the Cre gene is flanked by two homology boxes from the CCR2 gene and the first nucleotide of the CCR2 endogenous locus after the Cre stop codon is deleted (Fig 3A). The recombination cassette was cloned into the *AscI* and *NotI* sites of the pLD53.SC-AB shuttle vector [96], previously provided by Dr. D. Littman (NYU) [5]. The cassette was sequenced with overlapping primer sets to ensure the absence of mutations. For homologous recombination, the modified shuttle vector was electroporated into the BAC clone RP23-182D4. Clones that underwent BAC cointegration and resolution were isolated by chloramphenicol and ampicillin selection followed by sucrose negative selection, as previously described [5, 96]. Proper integration of the CCR2-Cre construct into the BAC was confirmed by Southern blot (S5A Fig) and sequencing of the modified regions. The CCR2-Cre BAC was then purified and injected into fertilized C57BL/6J oocytes by the University of Michigan Transgenic Animal Core. Four potential founder mice were identified out of 30 pups screened by PCR. These founders were bred to Rosa26^flSTOP-tdRFP^ mice and immune cells in the BM, blood, lungs and spleen were evaluated by flow cytometry. The progeny of two founders exhibited comparable tdRFP expression in a high percentage of monocytes (Fig 3B), and the lineage of one of these founders (#584) was chosen to establish the CCR2-Cre colony.

**Table 1:** Primers used in the generation of the CCR2-Cre mouse.

Primer Name	Sequence	Restriction Enzyme Sites
P1	5’-AACTTgACgCgTggCgCgCC***TCTAgA***AgCTAAAAgCAATATTTTTAAg-3’	MluI AscI ***XbaI***
P2	5’-CTCATCACTCgTggCgCCCATTTCCTTTgATTCTgTggTCAg-3'	
P3	5’-ATgggCgCCACgAgTgATgAg-3’	
P4	5’-CTAATCgCCATCTTCCAgCAg-3’	
P5	5’-CTgCTggAAgATggCgATTAgAAgACAATAATATgTTACCTC-3’	
P6	5’-gTAATTgCggCCgCgAATTCCTgAgTAgCAgATgAC-3’	NotI EcoRI

### Bone marrow chimeras

Bone marrow from STAT6^-/-^ mice (JAX stock #005977) [99] to generate chimeras was kindly supplied by Dr. P. Loke (NYU). Recipient CD45.1^+^ mice were exposed to 900 cGy in a cesium irradiator and then given 3–5 x 10^6^ donor STAT6^-/-^ or C57BL/6 (CD45.2^+^) BM cells by tail vein injection. Baytril 100 (enrofloxacin) was provided in the drinking water at a concentration of 0.4 mg/mL for the first 2 weeks after irradiation. Mice were used in experiments 6–8 weeks after irradiation.

### Infection with *Cryptococcus neoformans*

*C*. *neoformans* serotype A strain H99 #4413 was kindly provided by Dr J. Heitman (Duke). *C*. *neoformans* serotype D strain 52D (24067) was obtained from ATCC. All *C*. *neoformans* strains were maintained and grown as previously described [84]. Briefly, fungal strains were grown on Sabouraud dextrose agar (SAB) plates from frozen glycerol stocks and then cultured overnight at 37°C in liquid YPD medium (1% yeast extract, 2% peptone, 2% dextrose). Fungal cells were then washed and resuspended in phosphate-buffered saline (PBS) at a concentration of 10^3^ cells per 50 μl volume. Mice were anesthesized with inhaled isoflurane and given 50 μl of the fungal cell suspension intratracheally using a blunt-ended 20-gauge needle, as previously described [100].

### Analysis of infected mice

To assess fungal burden, murine lung and brain tissue were mechanically homogenized in PBS using a PowerGen 125 homogenizer (Fisher). Lymph nodes were dissociated using ground glass slides. CFU in all tissues were counted after plating serial dilutions of the homogenates on SAB plates.

To analyze cytokine levels, whole lungs were mechanically homogenized in 2 mL PBS containing cOmplete Protease Inhibitor Cocktail (Roche). ELISA assays were then performed on the supernatants from the homogenates using Ready-SET-Go ELISA kits (eBiosciences), except DuoSet ELISA Development Systems kits (R&D) were used to measure RANTES/CCL5 and IL-5.

For flow cytometry analysis, single cell lung suspensions were prepared as previously described [5] by digestion with DNase I (Roche) and collagenase type 4 (Worthington Biochemical) and mechanical disruption using a gentleMACS Dissociator (Miltenyi Biotec). Total lung cells were counted using a Coulter counter and stained with fluorescent antibodies. Flow cytometry data was collected on a BD LSR II flow cytometer and analyzed with FlowJo (v9.7.6). Monocyte progenitors are defined as Lin^-^(CD3ε ^-^CD19^-^NKp46^-^Sca-1^-^Ly6G^-^)CD45^+^CD117(c-Kit)^+^CD115^+^ with MDPs being Ly6C^lo^ and cMoPs being Ly6C^hi^. Inflammatory monocytes are CD45^+^Ly6G^-^MHCII^lo^CD11b^+^Ly6C^hi^. Ly6C^lo^ monocytes are CD45^+^Ly6G^-^MHCII^lo^CD11b^+^Ly6C^lo^. Macrophages are CD45^+^Ly6G^-^SiglecF^+^CD11c^hi^. DCs are CD45^+^Ly6G^-^CD11c^+^MHCII^hi^ and either CD11b^+^ or CD103^+^. Neutrophils are CD45^+^CD11b^+^Ly6G^+^. Eosinophils are CD45^+^Ly6G^-^SiglecF^+^CD11c^lo^. NK cells are Lin^-^(CD3ε^-^CD5^-^CD19^-^CD11c^-^)CD45^+^TcRβ^+^NK1.1^+^. ILCs are Lin^-^(CD3ε^-^CD5^-^CD8α^-^CD19^-^CD11c^-^CD11b^-^NK1.1^-^)CD45^+^CD90.2^+^CD127^+^. CD4^+^ T cells are CD45^+^CD3ε^+^CD90^+^CD4^+^. CD8^+^ T cells are CD45^+^CD4^-^CD19^-^CD8^+^. CD19^+^ B cells are CD45^+^CD4^-^CD8^-^CD19^+^. For additional gating information, see reference [84].

### Histopathology

The lungs of euthanized mice were perfused with 4% paraformaldehyde (PFA) in situ via a catheter inserted through an incision in the trachea. The lungs were then harvested and fixed by immersion in 4% PFA. Lungs were then processed by the MSKCC Molecular Cytology Core Facility to generate 4 μm sections of paraffin-embedded lungs stained with hematoxylin & eosin (H&E). Slides were scanned using a Zeiss Mirax Midi slide scanner with 20x/0.8NA objective. Slides were reviewed and scored by a pathologist. Morphometry analysis was carried out on scanned slide images using Pannoramic Viewer (v1.15.3, 3DHISTECH). Areas of lung inflammation were measured and expressed as a percentage of total lung area in each histologic section.

### Transcriptome analysis

Monocytes were pre-enriched from single cell lung suspensions pooled from 6–7 CCR2-GFP mice per group using the negative selection EasySep Mouse Monocyte Isolation Kit (STEMCELL Technologies). The enriched cells were then analyzed with a BD FACSAria cell sorter to obtain DAPI^-^Lin^-^CD11b^+^Ly6C^hi^CCR2^GFP^ cells (Lin^-^ = Ter119^-^CD3^-^CD19^-^NK1.1^-^CD11c^-^Ly6G^-^). RNA was extracted from sorted cells using TRIzol LS (Thermo Fisher Scientific) according to the manufacturer’s instructions.

RNASeq was performed by the MSKCC Integrated Genomics Operation. After RiboGreen quantification and quality control by an Agilent BioAnalyzer, 500ng of total RNA underwent polyA selection and TruSeq library preparation according to instructions provided by Illumina (TruSeq Stranded mRNA LT Kit, catalog # RS-122-2102), with 8 cycles of PCR. Samples were barcoded and run on a HiSeq 2500 in a 50bp/50bp paired end run, using the TruSeq SBS Kit v4 (Illumina). An average of 44.6 million paired reads was generated per sample. At the most the ribosomal reads represented 0.01% of the total reads generated and the percent of mRNA bases averaged 73.5%. The RNASeq data have been deposited in NCBI’s Gene Expression Omnibus (GEO) [101] and are accessible through GEO Series accession number GSE122765 (https://www.ncbi.nlm.nih.gov/geo/query/acc.cgi?acc=GSE122765).

Statistical analysis of RNASeq data was performed by the MSKCC Bioinformatics Core. The output data (FASTQ files) were mapped to the target genome using the rnaStar aligner [102] that maps reads genomically and resolves reads across splice junctions. The 2 pass mapping method [103] was used, in which the first mapping pass uses a list of known annotated junctions from Ensembl. Novel junctions found in the first pass were then added to the known junctions and a second mapping pass was performed in which the RemoveNoncanoncial flag was used. After mapping, the output SAM files were post processed using the PICARD tool AddOrReplaceReadGroups to add read groups, sort the files, and covert them to the compressed BAM format. The expression count matrix was then computed from the mapped reads using HTSeq (www-huber.embl.de/users/anders/HTSeq) and one of several possible gene model databases. The raw count matrix generated by HTSeq was then processed using the R/Bioconductor package DESeq (www-huber.embl.de/users/anders/DESeq) which was used to both normalize the full dataset and analyze differential expression between sample groups.

For quantitative RT-PCR, cDNA was generated from RNA using a QuantiTect Reverse Transcription Kit (Qiagen), and qRT-PCR was performed on a StepOnePlus Real Time PCR System (Applied Biosystems) using TaqMan Fast Advanced Master Mix and TaqMan Gene Expression Assays (ThermoFisher Scientific) including Arg1 (Mm00475988_m1), Mrc1 (Mm01329362_m1), Retnla/Fizz1 (Mm00445109_m1), Hprt (Mm03024075_m1), Nos2 (Mm00440502_m1), Tnf (Mm00443258_m1), Chil3/Ym1 (Mm00657889_mH) Zbtb46 (Mm00511327_m1), and Mafb (Mm00627481_s1).

### Statistical analysis

All results are expressed as mean ± SEM. A Mann-Whitney U test was used for statistical analysis of two group comparisons, and one-way ANOVA was used for three groups or more, unless otherwise noted. Survival data was analyzed by Mantel-Cox test. All statistical analyses were performed with GraphPad Prism software, v6.0f. A *P* value < 0.05 was considered significant and indicated with an asterisk.

## Supporting information

S1 Fig Murine model of acute infection with *C. neoformans* strain H99. (A) Kaplan-Meier survival curves of C57BL/6 (WT) mice infected with 10^2^−10^5^ H99 yeast cells i.t. There was no significant difference between the survival of mice given 10^5^ and 10^4^ of H99. Mice given 10^3^ and 10^2^ of H99 had significant improvements in survival compared to the 10^5^ and 10^4^ inocula, with *P* values shown relative to 10^4^ H99. Data were pooled from two experiments (*n* = 7–15 mice per group). (B) CD11b^+^ DCs and macrophages in the lungs of mice infected with 10^3^ H99 compared to naive mice (dotted line). Data were pooled from eight experiments (*n* = 3–26 mice per timepoint). *, *P* < 0.05. **, *P* < 0.01. ****, *P* < 0.0001.
(TIF)

S2 Fig Profile of immune cells in the lungs after monocyte ablation. Immune cell populations in the lungs of WT mice (white circles) and IM-ablated CCR2-DTR mice (black circles) on days 7 and 14 after i.t. challenge with H99. Data were pooled from four independent experiments (*n* = 8–14 total mice per group). **, *P* < 0.01. ***, *P* < 0.001. ****, *P* < 0.0001.
(TIF)

S3 Fig Inflammatory monocytes regulate the proliferation of different *C. neoformans* serotypes in the lungs but do not regulate outcomes at later stages of infection. (A-B) CFU in the lungs of WT mice (white circles) or IM-ablated CCR2-DTR mice (black circles) challenged i.t. with 10^4^ yeast cells of (A) serotype A strain H99 on day 14 p.i. and (B) serotype D strain 52D on day 7 p.i. Data are from one experiment per serotype (*n* = 3–4 mice per group). (C) Kaplan-Meier survival curves of WT mice (white circles) and CCR2-DTR mice (black circles) given DT on days +6, +8, and +10 after i.t. challenge with 10^3^ H99 yeast cells. Data are from one experiment (*n* = 4–5 mice per group). *, *P* < 0.05 by *t*-test.
(TIF)

S4 Fig Ablation of inflammatory monocytes reduces lung size, pulmonary infiltrates, and intracellular organisms. (A) Whole lungs from WT and IM-ablated CCR2-DTR mice on day 26 p.i. with H99. (B) Representative H&E stained sections of the lungs from WT and CCR2-DTR mice on day 14 p.i. and measurement of pulmonary infiltrates (example of an area of infiltration outlined in black) (scale bar = 2000 μm). Data were pooled from two independent experiments (*n* = 6 total mice per group). (C) Representative areas of infiltrates in H&E stained lung sections of WT and CCR2-DTR mice on day 14 p.i. (scale bar = 50 μm), showing an eosinophil predominance. *C*. *neoformans* cells (black arrows) are visualized within multinucleated giant cells (MGC, white asterisks) in WT lungs. (D) Scoring of histologic sections. Data are from one experiment (*n* = 5 mice per group). *, *P* < 0.05 and **, *P* < 0.01 by *t*-test.
(TIF)

S5 Fig Validation of the CCR2-Cre mouse. (A) Southern blot using a Cre probe of a Cre-containing plasmid (+), the unmodified BAC (-), and the CCR2-Cre BAC (CC) digested with BspHI or HindIII to confirm appropriate integration of the CCR2-Cre construct. (B-G) Representative flow plots and histograms of tdRFP expression in naive CCR2-Cre Rosa26^flSTOP-tdRFP^ mice by (B) monocyte progenitors in BM, (C) macrophages and CD103^+^ DCs in the lungs, (D) CD11b^+^ DCs, (E) Ly6C^lo^ Mo, (F) neutrophils, and (G) lymphocytes. Dotted lines in histograms represent naive Rosa26^flSTOP-tdRFP^ control mice.
(TIF)

S6 Fig Inflammatory monocytes modulate cytokines in the lungs. Lung cytokine levels were measured by ELISA in WT mice (white bars) and IM-ablated CCR2-DTR mice (gray bars) on day 7 p.i. with H99. Measurements below the limit of detection were recorded as zero. Data were pooled from 3 independent experiments (*n* = 5–9 total mice per group). *, *P* < 0.05. ***, *P* < 0.001.
(TIF)

S7 Fig Validation of the conditional knockout of MHCII expression. (A) Representative flow plots, (B) histogram, and (C) quantitation by mean fluorescence intensity (MFI) of MHCII expression by CD45^+^Ly6G^-^SiglecF^-^CD103^-^CD11b^+^CD11c^+^ lung cells from CCR2-Cre MHCII^fl/fl^ mice (red line and black circles) and control MHCII^fl/fl^ mice (dotted line and white circles) on day 14 p.i. with H99. Data are from one experiment (*n* = 4–6 mice per group). **, *P* < 0.01.
(TIF)

S8 Fig Conditional knockout of arginase 1 expression. (A) Arginase activity in the lysate of 10^6^ lung cells from Arg1^fl/fl or fl/+^ mice (white circles) and CCR2-Cre Arg1^fl/fl^ mice (black circles) on day 14 p.i. with H99. Data were from one experiment (*n* = 5 mice per group). **, *P* < 0.01. (B) Kaplan-Meier survival curve of Arg1^fl/fl or fl/+^ mice and Vav1-Cre Arg1^fl/fl^ mice (gray circles) challenged with H99. Data were from one experiment (*n* = 5 mice per group).
(TIF)
