# Supplementary figures and images for: Inflammatory monocytes are detrimental to the host immune response during acute infection with Cryptococcus neoformans
Source: PLoS Pathog. 2019 Mar 21;15(3):e1007627. doi: 10.1371/journal.ppat.1007627 (PMC6428256; doi:10.1371/journal.ppat.1007627)

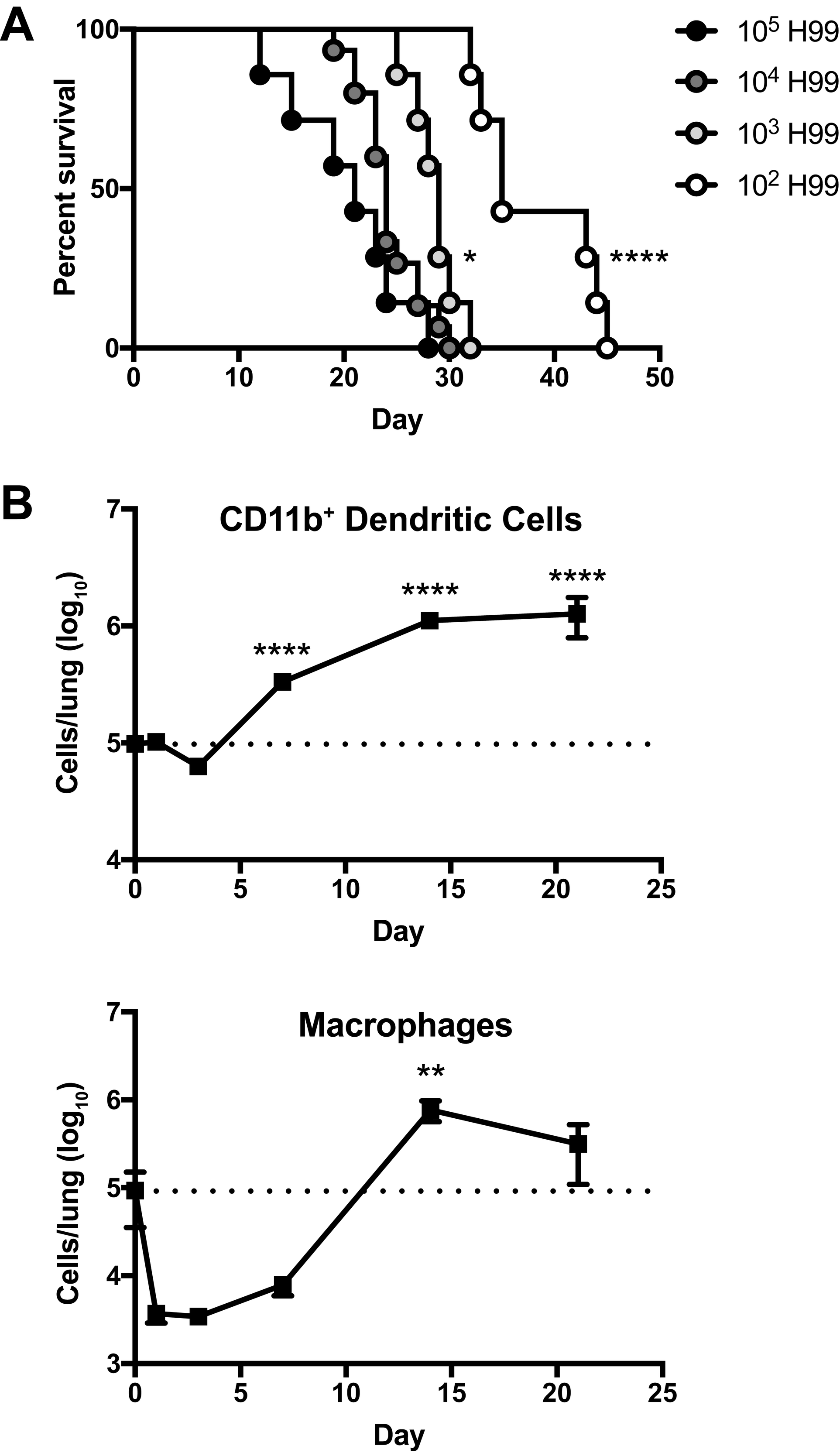

Supplement: S1 Fig — (A) Kaplan-Meier survival curves of C57BL/6 (WT) mice infected with 102−105 H99 yeast cells i.t. There was no significant difference between the survival of mice given 105 and 104 of H99. Mice given 103 and 102 of H99 had significant improvements in survival compared to the 105 and 104 inocula, with P values shown relative to 104 H99. Data were pooled from two experiments (n = 7–15 mice per group). (B) CD11b+ DCs and macrophages in the lungs of mice infected with 103 H99 compared to naive mice (dotted line). Data were pooled from eight experiments (n = 3–26 mice per timepoint). *, P < 0.05. **, P < 0.01. ****, P < 0.0001. (TIF) [file ppat.1007627.s001.tif]

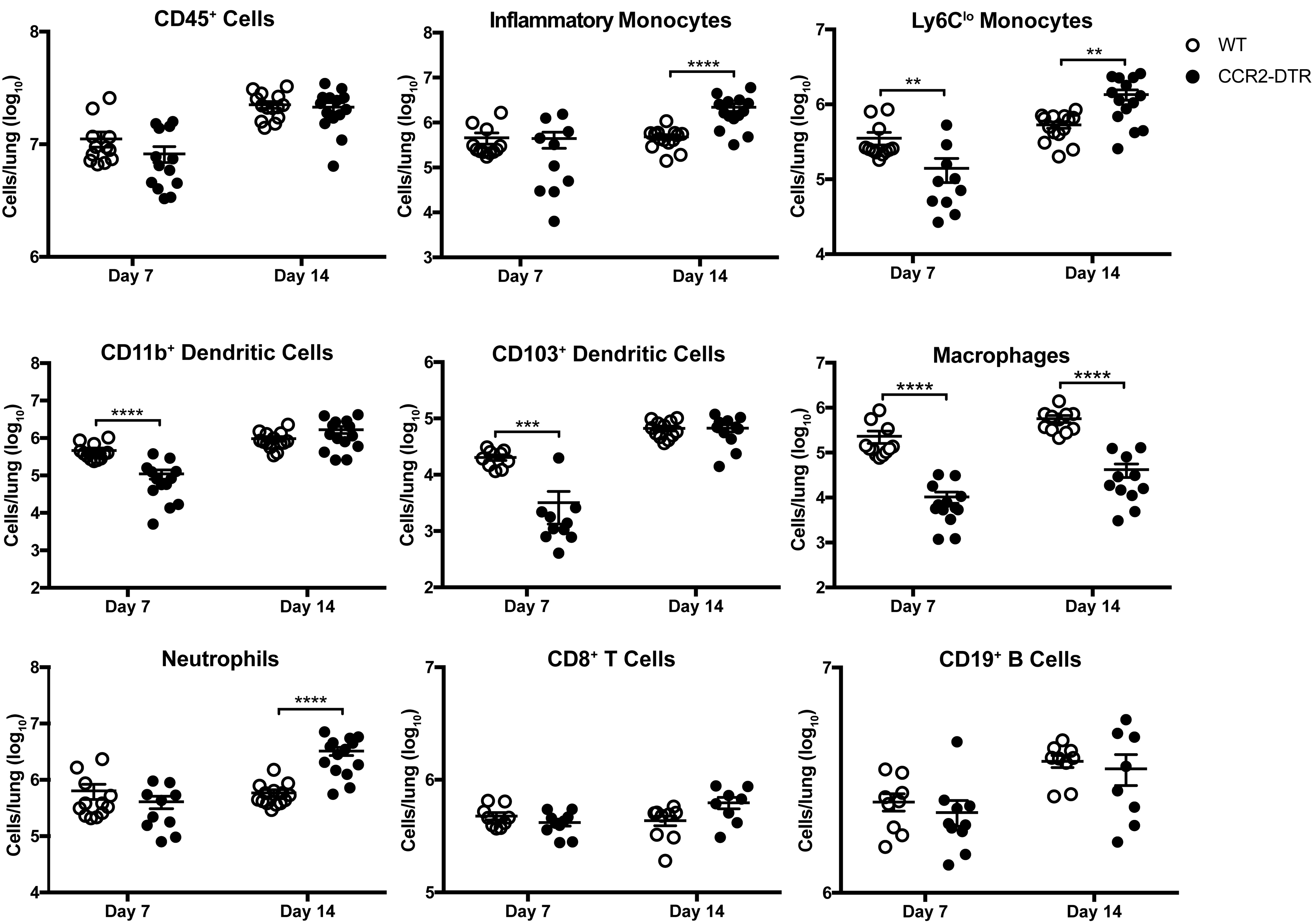

Supplement: S2 Fig — Immune cell populations in the lungs of WT mice (white circles) and IM-ablated CCR2-DTR mice (black circles) on days 7 and 14 after i.t. challenge with H99. Data were pooled from four independent experiments (n = 8–14 total mice per group). **, P < 0.01. ***, P < 0.001. ****, P < 0.0001. (TIF) [file ppat.1007627.s002.tif]

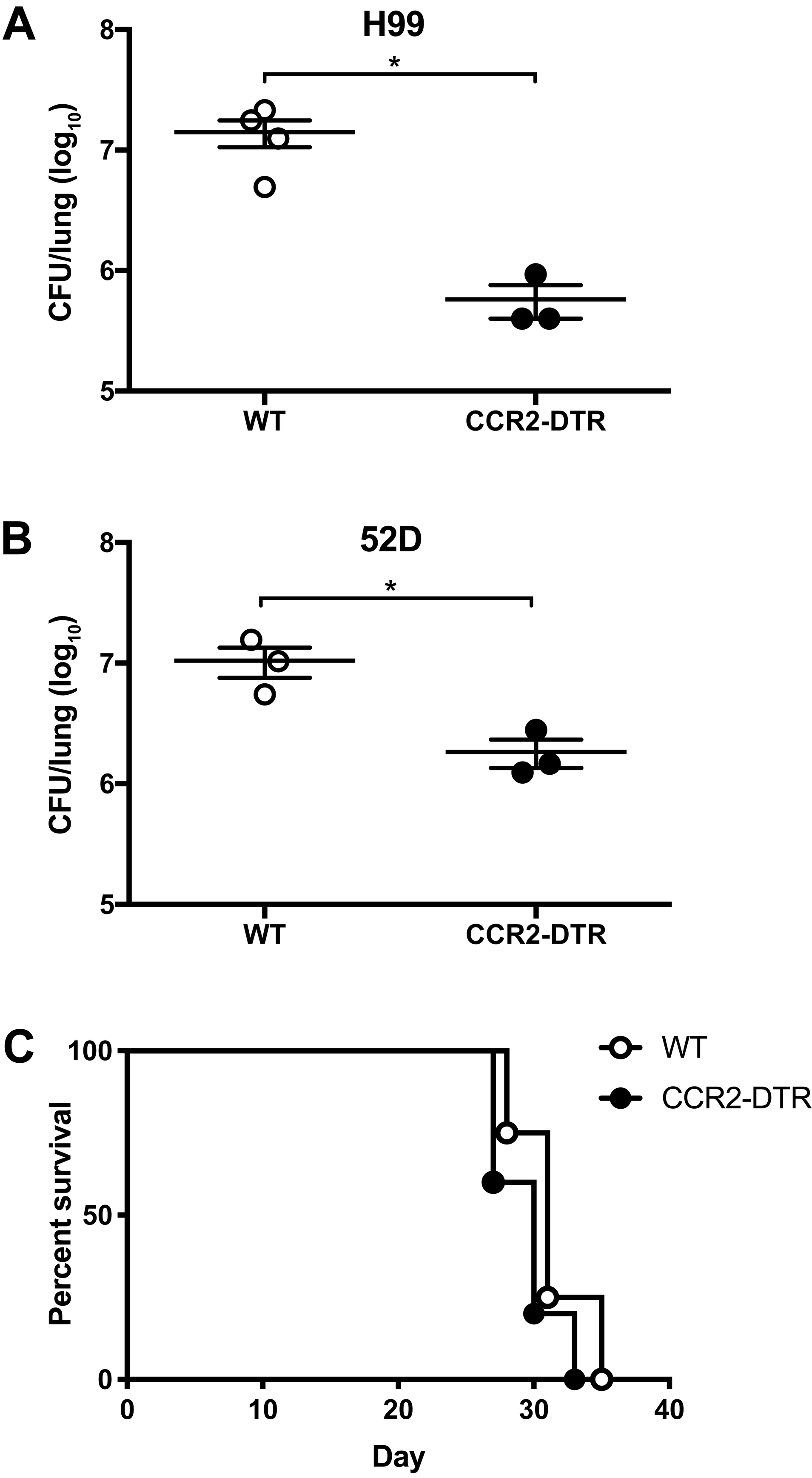

Supplement: S3 Fig — (A-B) CFU in the lungs of WT mice (white circles) or IM-ablated CCR2-DTR mice (black circles) challenged i.t. with 104 yeast cells of (A) serotype A strain H99 on day 14 p.i. and (B) serotype D strain 52D on day 7 p.i. Data are from one experiment per serotype (n = 3–4 mice per group). (C) Kaplan-Meier survival curves of WT mice (white circles) and CCR2-DTR mice (black circles) given DT on days +6, +8, and +10 after i.t. challenge with 103 H99 yeast cells. Data are from one experiment (n = 4–5 mice per group). *, P < 0.05 by t-test. (TIF) [file ppat.1007627.s003.tif]

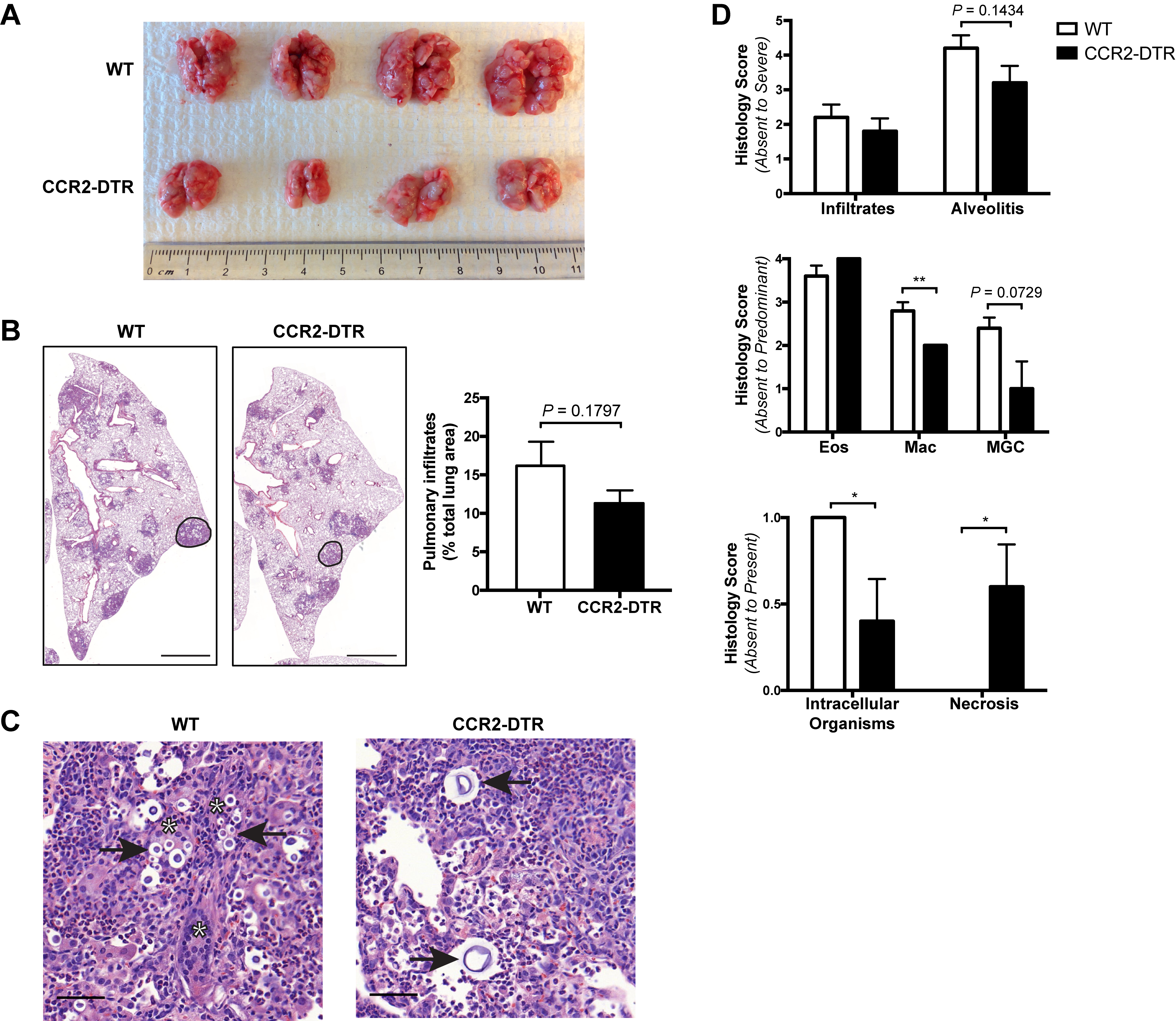

Supplement: S4 Fig — (A) Whole lungs from WT and IM-ablated CCR2-DTR mice on day 26 p.i. with H99. (B) Representative H&E stained sections of the lungs from WT and CCR2-DTR mice on day 14 p.i. and measurement of pulmonary infiltrates (example of an area of infiltration outlined in black) (scale bar = 2000 μm). Data were pooled from two independent experiments (n = 6 total mice per group). (C) Representative areas of infiltrates in H&E stained lung sections of WT and CCR2-DTR mice on day 14 p.i. (scale bar = 50 μm), showing an eosinophil predominance. C. neoformans cells (black arrows) are visualized within multinucleated giant cells (MGC, white asterisks) in WT lungs. (D) Scoring of histologic sections. Data are from one experiment (n = 5 mice per group). *, P < 0.05 and **, P < 0.01 by t-test. (TIF) [file ppat.1007627.s004.tif]

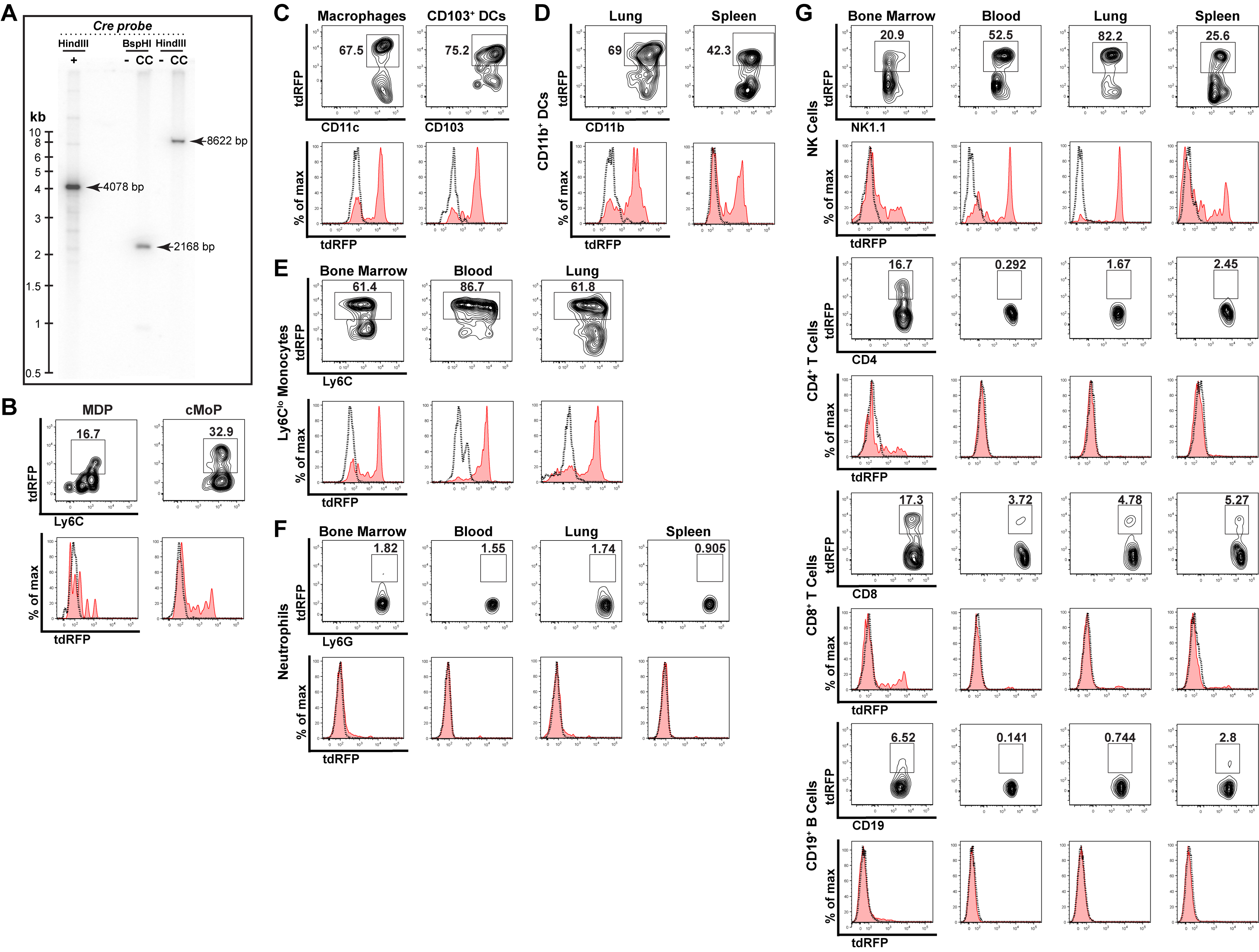

Supplement: S5 Fig — (A) Southern blot using a Cre probe of a Cre-containing plasmid (+), the unmodified BAC (-), and the CCR2-Cre BAC (CC) digested with BspHI or HindIII to confirm appropriate integration of the CCR2-Cre construct. (B-G) Representative flow plots and histograms of tdRFP expression in naive CCR2-Cre Rosa26flSTOP-tdRFP mice by (B) monocyte progenitors in BM, (C) macrophages and CD103+ DCs in the lungs, (D) CD11b+ DCs, (E) Ly6Clo Mo, (F) neutrophils, and (G) lymphocytes. Dotted lines in histograms represent naive Rosa26flSTOP-tdRFP control mice. (TIF) [file ppat.1007627.s005.tif]

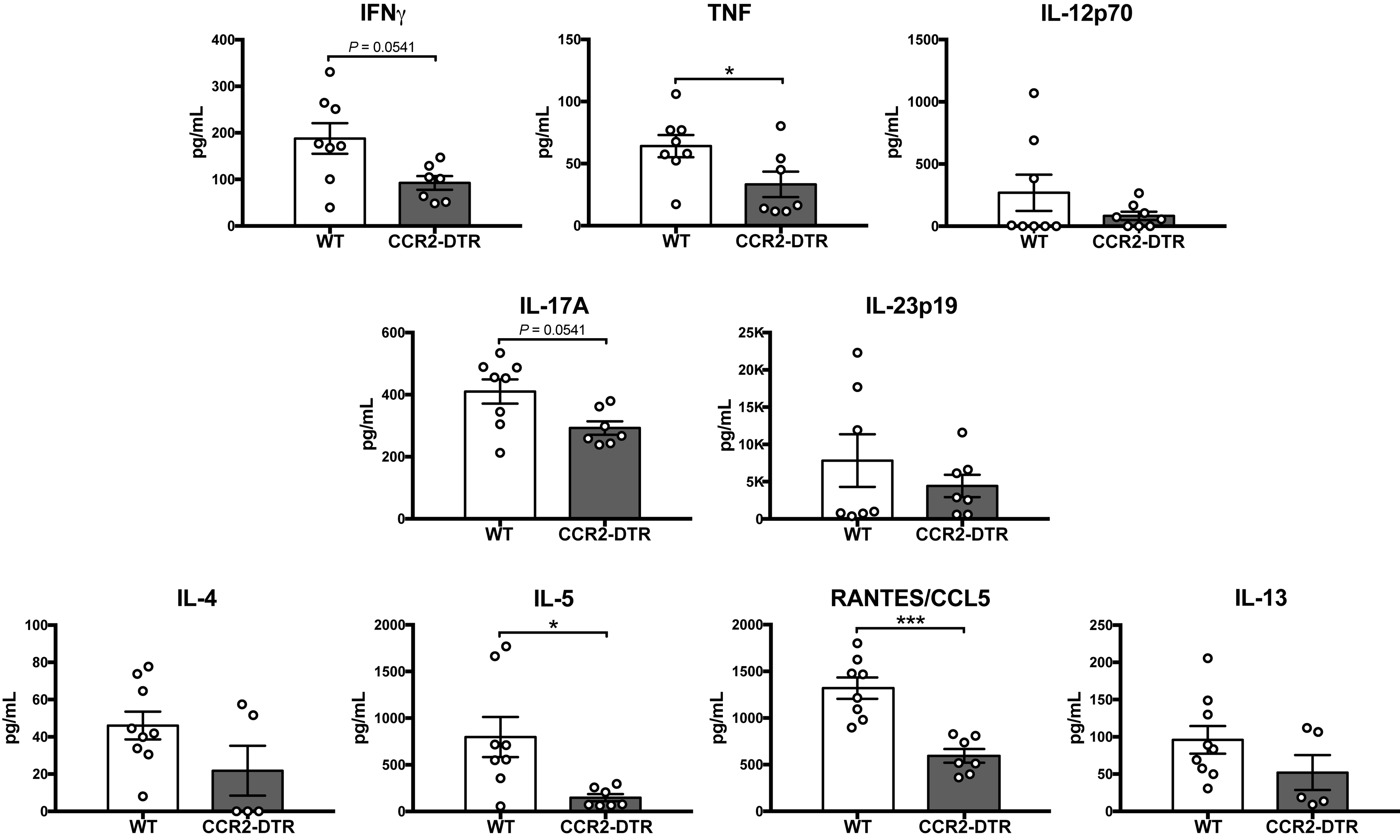

Supplement: S6 Fig — Lung cytokine levels were measured by ELISA in WT mice (white bars) and IM-ablated CCR2-DTR mice (gray bars) on day 7 p.i. with H99. Measurements below the limit of detection were recorded as zero. Data were pooled from 3 independent experiments (n = 5–9 total mice per group). *, P < 0.05. ***, P < 0.001. (TIF) [file ppat.1007627.s006.tif]

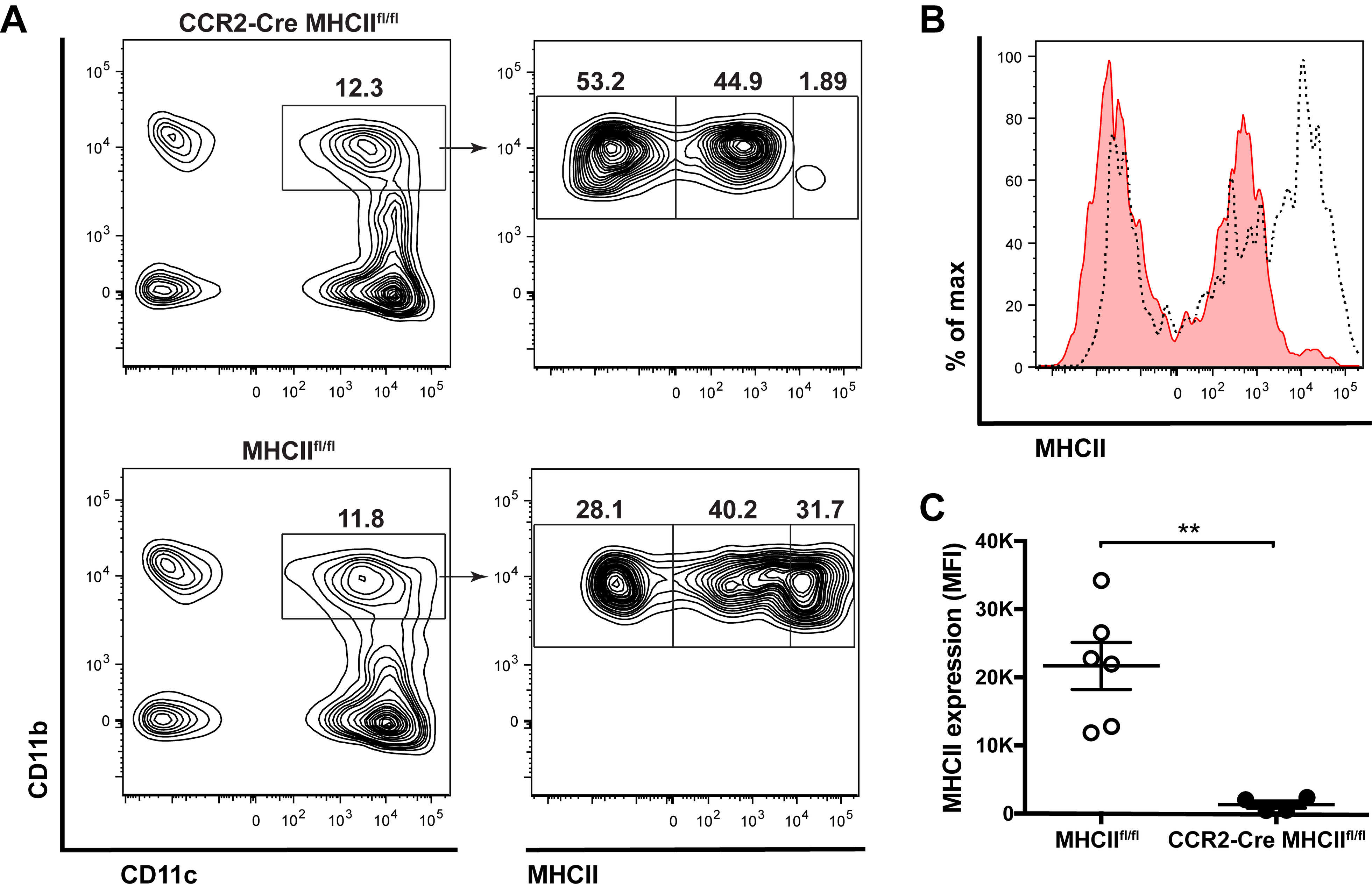

Supplement: S7 Fig — (A) Representative flow plots, (B) histogram, and (C) quantitation by mean fluorescence intensity (MFI) of MHCII expression by CD45+Ly6G-SiglecF-CD103-CD11b+CD11c+ lung cells from CCR2-Cre MHCIIfl/fl mice (red line and black circles) and control MHCIIfl/fl mice (dotted line and white circles) on day 14 p.i. with H99. Data are from one experiment (n = 4–6 mice per group). **, P < 0.01. (TIF) [file ppat.1007627.s007.tif]

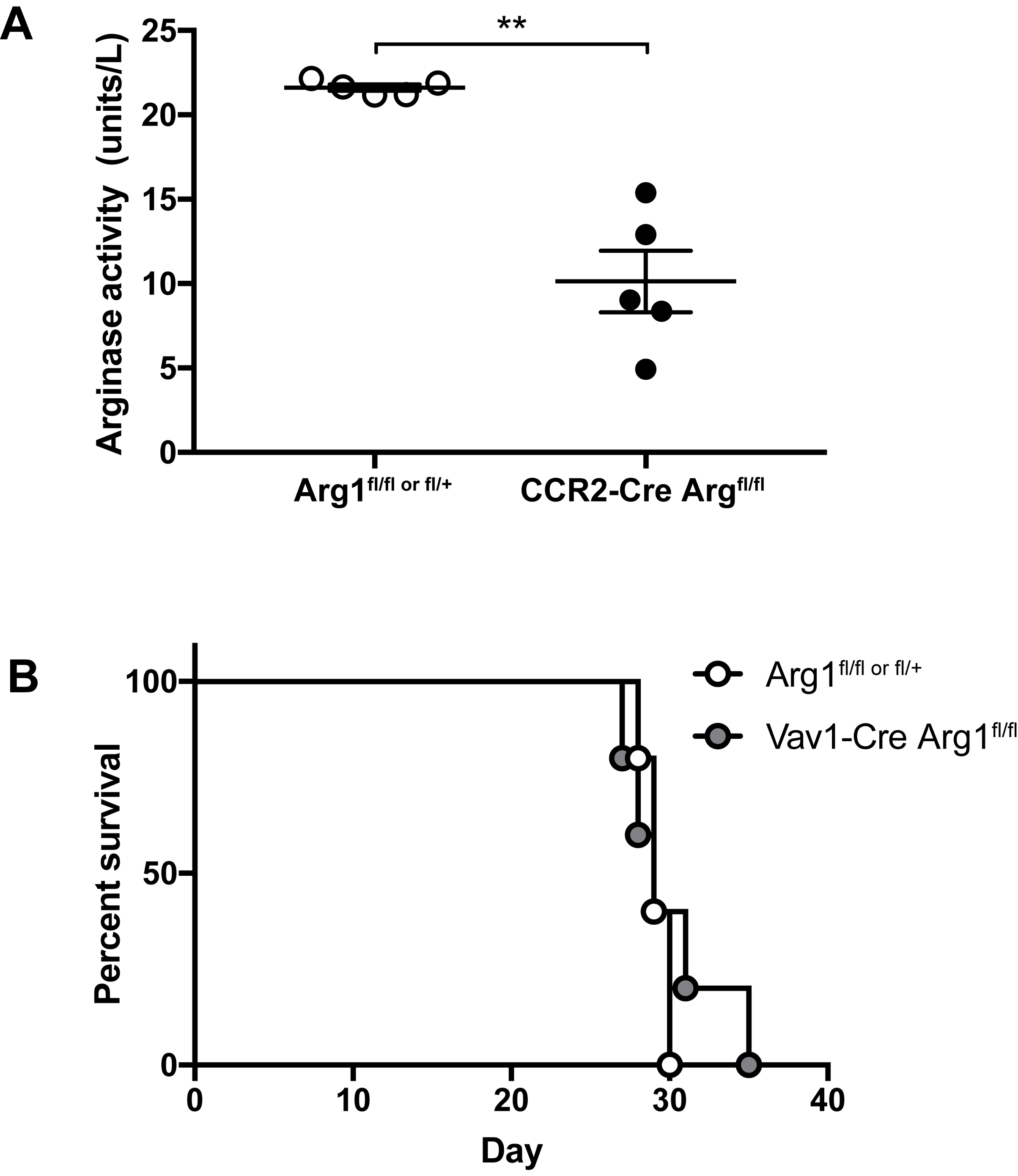

Supplement: S8 Fig — (A) Arginase activity in the lysate of 106 lung cells from Arg1fl/fl or fl/+ mice (white circles) and CCR2-Cre Arg1fl/fl mice (black circles) on day 14 p.i. with H99. Data were from one experiment (n = 5 mice per group). **, P < 0.01. (B) Kaplan-Meier survival curve of Arg1fl/fl or fl/+ mice and Vav1-Cre Arg1fl/fl mice (gray circles) challenged with H99. Data were from one experiment (n = 5 mice per group). (TIF) [file ppat.1007627.s008.tif]
